# Polyphenol-rich diet mediates interplay between macrophage-neutrophil and gut microbiota to alleviate intestinal inflammation

**DOI:** 10.1038/s41419-023-06190-4

**Published:** 2023-10-09

**Authors:** Dandan Han, Yujun Wu, Dongdong Lu, Jiaman Pang, Jie Hu, Xiangyu Zhang, Zhenyu Wang, Guolong Zhang, Junjun Wang

**Affiliations:** 1 State Key Laboratory of Animal Nutrition and Feeding, College of Animal Science and Technology, China Agricultural University, Beijing, 100193 China; 2 Department of Animal and Food Sciences, Oklahoma State University, Stillwater, OK 74078 USA

**Keywords:** Inflammatory bowel disease, Mucosal immunology, Cell polarity

## Abstract

Dietary phenolic acids alleviate intestinal inflammation through altering gut microbiota composition and regulating macrophage activation. However, it is unclear how individual phenolic acids affect the interactions between intestinal microbiota and macrophages in the context of inflammatory bowel disease (IBD). Here, we aim to elucidate the mechanism by which phenolic acids alleviate gut inflammation. Mice with or without depletion of macrophages were administered with four individual phenolic acids including chlorogenic, ferulic, caffeic, and ellagic acids, following dextran sulfate sodium (DSS) treatment. Gut microbiota depletion and fecal microbiota transplantation were further performed in mice to investigate the role of the gut microbiota in phenolic acid-mediated protective effect. Colitis severity was evaluated using histological, serological, and immunological measurements. Absence of intestinal microbiota and macrophage deteriorate the epithelial injury in DSS colitis. Chlorogenic acid mitigated colitis by reducing M1 macrophage polarization through suppression of pyruvate kinase M 2 (Pkm2)-dependent glycolysis and inhibition of NOD-like receptor protein 3 (Nlrp3) activation. However, ferulic acid-mediated reduction of colitis was neutrophil-dependent through diminishing the formation of neutrophil extracellular traps. On the other hand, the beneficial effects of caffeic acid and ellagic acid were dependent upon the gut microbiota. In fact, urolithin A (UroA), a metabolite transformed from ellagic acid by the gut microbiota, was found to alleviate colitis and enhance gut barrier function in an IL22-dependent manner. Overall, our findings demonstrated that the mechanisms by which phenolic acid protected against colitis were resulted from the interaction between gut microbiota and macrophage-neutrophil.

## Introduction

Inflammatory bowel disease (IBD) is caused by hyperactive and dysregulated mucosal immune response to resident microbiota, resulting in intestinal inflammation, tissue damage, and microbial dysbiosis [1, 2]. Although the precise etiopathogenesis of IBD is still unclear, a growing body of evidence suggests a central role of macrophages in the pathogenesis of IBD [3–7]. Macrophages possess the remarkable plasticity for further differentiation, maintenance of intestinal homeostasis, and bidirectional regulation of inflammation [5, 8]. Macrophages undergo rapid changes in morphology and activation status to adapt to a specific milieu, following stimulation with microbial products or host cytokines [9]. IBD may arise from dysregulated proinflammatory macrophage activation in response to an alteration in the gut microbiota [5]. A shift from the inflammatory to regulatory immune phenotype of macrophages contributes to the alleviation of colitis by enhancing M2 polarization, restraining IL-6 production in M1 macrophages, and changing the gut homeostasis [3, 6, 7]. Notably, recent studies have shown an association of the metabolic profile with the phenotype and function of macrophages [10–12]. Therefore, precise regulation of aberrant macrophage polarization and immunometabolism is of paramount importance in the clearance of microorganisms, elimination of damaged cells, and production of mediators that drive epithelial cell renewal.

Gut microbiota is well known to be critically involved in maintaining the integrity of the gastrointestinal tract, the gut mucosal homeostasis, gastrointestinal cancer, and host nutritional metabolism [13–17]. Alterations in the composition of gut microbes and microbial metabolites were recently demonstrated to be associated with IBD [18–21]. Intestinal microbiota is required for colitis initiation and development in mice treated with dextran sodium sulfate (DSS)-treated mice or deficient in IL-10 [22–25]. Restoring the gut homeostasis through normalization of the gut microbiota is considered a viable target for therapeutic intervention in IBD. In fact, transplantation of the gut microbiota from healthy donors to colitis recipients has been shown to alleviate the inflammatory response in a murine colitis model [22]. We previously reported that transplantation of fecal microbiota from green tea polyphenolic-dosed mice improved intestinal epithelial homeostasis and ameliorated colitis by altering the microbial community structure and metabolic profile [26].

Phenolic acids constitute a large group of compounds containing a phenolic ring and a carboxylic acid group. Dietary phenolic acids have been shown to improve gut function by reducing intestinal inflammation and altering the gut microbiota [26–29]. Furthermore, polyphenols and the microbiota work in concert to impact intestinal inflammation through regulating macrophage activation and gut microbial metabolites [26, 29–31]. Especially consumption of diets containing chlorogenic, ferulic, caffeic, and ellagic acids, critically influenced gut health by shaping the gut microbiota composition and regulating immune homeostasis [27, 32–35]. Presently, little is known about the involvement of the gut microbiota and macrophages in phenolic acid-mediated alleviation of gut inflammation. To understand the complex interactions between the microbiota and macrophages in a controlled manner, we used either antibiotic-treated or macrophage-deleted mice combined with four unique phenolic acids to investigate the interplay of phenolic acids with the gut microbiota and macrophages.

## Results

### Intestinal microbiota-macrophage crosstalk influences the colitis severity

**Fig. 1:**
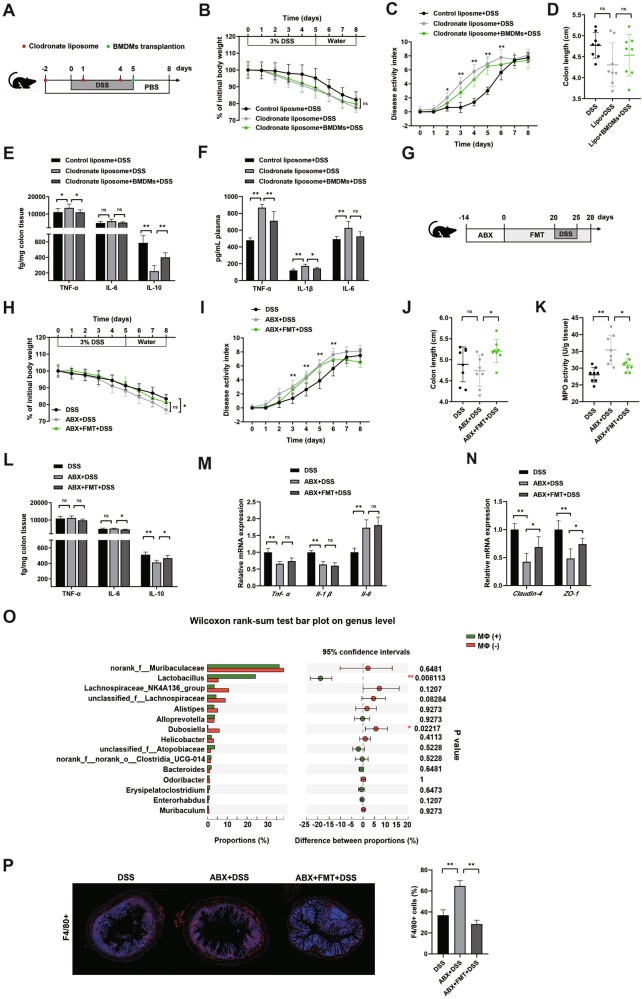
Absence of intestinal microbiota and macrophage enhances epithelial injury in DSS colitis. **A** Mice were intraperitoneally injected with 200 μL clodronate-loaded liposomes three times every 3 days before 2 days giving 3% DSS in drinking water, and also on day 1 and day 4 during DSS treatment. Some macrophage-depleted mice were received BMDMs isolated from WT mice before DSS treatment. **B**–**D** Colitis severity was measured as body weight loss, DAI, and colon length (*n* = 8). **E, F** The levels of TNF-α, IL-6 and IL-10 in colon homogenates, and secretion of TNF-α, IL-1β and IL-6 in serum. **G** Mice received four-ABX cocktail in the drinking water of the DSS colitis model. Fecal microbial suspension from individual donors was transplanted into some antibiotic-treated mice before giving 3% DSS in drinking water. **H**–**K** Colitis severity was measured as body weight loss, DAI, colon length, and MPO activity (*n* = 8). **L** The levels of TNF-α, IL-6 and IL-10 in colon homogenates. **M, N** The mRNA levels of *Tnf-α*, *Il-1β*, *Il-6*, *Claudin-4*, and *ZO-1* in colonic tissue. **O** The microbiota composition of macrophage-depleted mice. *P* values were FDR corrected. *P < 0.05, **P < 0.01; Wilcoxon rank-sum test. **P** The infiltration of F4/80+ positive macrophage in antibiotic-treatment DSS colitis mice. Data represent means ± SD of three separate experiments. Statistics was performed with one-way ANOVA, followed by Tukey’s multiple comparison test. *P < 0.05, **P < 0.01.

To investigate the interplay between gut microbiota and macrophage during intestinal inflammation, we induced colitis in macrophage-depleted mice implanted with BMDMs from WT mice, and in antibiotic-treated mice conventionalized with the microbiota from SPF mice, respectively. We depleted macrophage by in vivo administration of clodronate-loaded liposomes on 2 days before 3% DSS treatment, and also on day 1 and day 4 during DSS treatment (Fig. 1A). Compared to 28.23 ± 2.41% in the control group, the F4/80 positive cells were significantly increased in clodronate-loaded liposomes-injected mice (57.65 ± 4.84%) (Supplementary Fig. 1A). Mice with macrophage deletion are more susceptible to DSS-induced colitis that measured by increased disease activity index (DAI) (Fig. 1C), but not weight loss (Fig. 1B), and colon length (Fig. 1D). To determine the immunological basis of increased colitis in macrophage-depleted mice, cytokines production in the colon were analyzed. The secretion of TNF-a but not IL-6 was profoundly increased in the colon of macrophage-depleted mice after DSS administration. The reduced IL-10 level was observed in macrophage-deficient mice (Fig. 1E). Consistently, multi-cytokine analysis showed that macrophage ablation induced higher serum inflammatory factors levels in DSS-treated mice (Fig. 1F). This indicated that restoring macrophage population in depleted mice reduces cytokine levels but does not ameliorate disease severity. To further study the role of macrophage during intestinal inflammation, we implanted the BMDMs from healthy donors into macrophage-depleted colitis mice on day 5 during DSS treatment (Fig. 1A). The recipients showed the reduced secretion of TNF-α and IL-1β, and increased IL-10 level (Fig. 1E, F), indicating that the absence of macrophage promoted intestinal inflammation.

Mice were subjected to oral inoculation with fecal microbial suspensions from individual healthy donors, administered once every other day for 4 weeks after receiving a four-antibiotics cocktail in the drinking water for 2 weeks (Fig. 1G). As expected, the mice exhibited an obvious decrease in bacterial DNA content (Supplementary Fig. 1B) with a 4-log reduction in total bacterial population within the fecal samples (Supplementary Fig. 1C). The bacterial DNA concentration and total bacteria of FMT mice receiving antibiotic administration were significantly greater than that of antibiotic-treated mice in the feces (Supplementary Fig. 1B, C). Mice subjected to antibiotic treatment displayed heightened susceptibility to DSS-induced colitis as shown in the increased weight loss (Fig. 1H), disease activity indices (Fig. 1I), and higher colonic myeloperoxidase (MPO) activity (Fig. 1K), but less prone to inflammatory response than SPF colitis mice, as evidenced by the lower secretion of IL-10 in colon tissue (Fig. 1L), up-regulated *Il-6* gene level, and the down-regulated *Tnf-α* and *Il-1β* expression (Fig. 1M). Meanwhile, microbiota-depleted mice suffered exacerbated epithelial injury as a consequence of a weakened intestinal barrier, as shown by lower mRNA levels of *Claudin-4* and *ZO-1* (Fig. 1N). To further determine the presence of gut microbiome associated with colitis progression, we transplanted the microbiota from SPF mice into microbiota-depleted colitis recipients. Mice receiving fecal microbiota from controls exhibited increased colon length (Fig. 1J), decreased MPO activity (Fig. 1K), and enhanced gene expression related to gut barrier function (Fig. 1N). FMT mice also showed a lower secretion of IL-6 but the higher secretion of IL-10 in colonic tissue (Fig. 1L). However, FMT failed to rescue the percentage of weight loss (Fig. 1H) and the mRNA levels of *Tnf-α*, *Il-1β*, and *Il-6* (Fig. 1G), suggesting that intestinal microbiota is involved in the development of DSS colitis. In addition, we also demonstrated that depletion of macrophage altered microbiome composition (Fig. 1I, and Supplementary Fig. 2). The increased population of F4/80 positive macrophage was reduced by FMT in microbiota-deleted mice (Fig. 1P), indicating that the gut microbiome could influence the infiltration of macrophage during DSS colitis. These findings confirmed that the interplay between gut microbiome and macrophage is associated with the maintenance of epithelial inflammatory injury.

### Phenolic acids ameliorate colitis and alter the macrophage phenotype

**Fig. 2:**
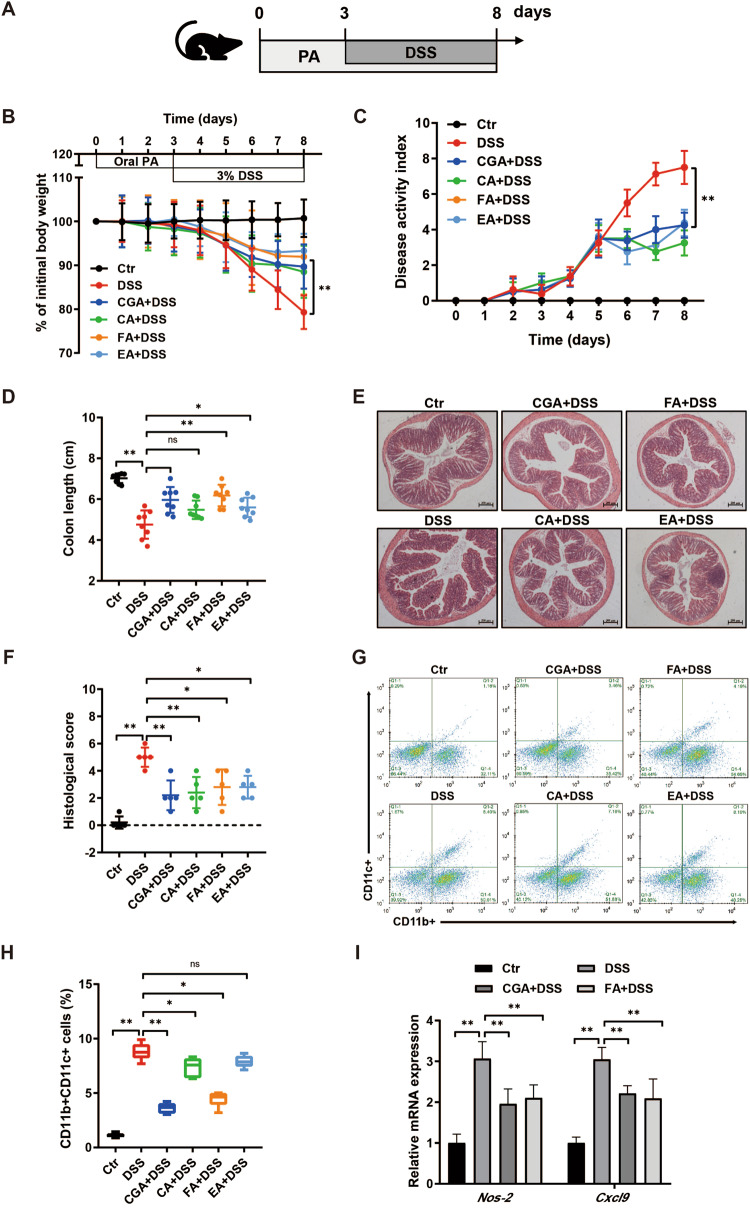
Phenolic acids alleviate DSS-induced colitis potentially shifting macrophage polarization. **A** Mice were administrated with four unique phenolic acids, chlorogenic acid, caffeic acid, ferulic acid and ellagic acid for 8 days, and on day 3, acute experimental colitis was induced with 3% DSS in drinking water given ad libitum for 5 days. **B**–**D** Colitis severity was measured as body weight loss, DAI, and colon length (*n* = 8). **E, F** H&E staining of colon tissue and histological scores at day 8 after oral phenolic acids. **G, H** Flow cytometry analysis of pro-inflammatory CD11b + CD11c+ colonic macrophage. **I** The mRNA induction of M1 macrophage signature genes *Nox2*, *Cxcl9* in colonic tissue. Data represent means ± SD of three separate experiments. Statistics was performed with one-way ANOVA, followed by Tukey’s multiple comparison test. *P < 0.05, **P < 0.01.

Given that the intestinal microbiota-macrophage crosstalk, we investigate the effect of phenolic acids on colitis and whether this impact depends on the presence of gut microbiome and macrophage. To study the beneficial effect of phenolic acids, mice were orally administrated with four unique phenolic acids for 8 days, and on day 3, 3% DSS was provided in drinking water ad libitum for 5 days to induce acute experimental colitis, as illustrated in Fig. 2A. Chlorogenic acid was effective against colitis, to include effects in weight loss, DAI, colon length, as well as intestinal pathology and histology score (Fig. 2B–F). Similarly, caffeic acid, ferulic acid, and ellagic acid were also able to significantly ameliorate the severity of colitis, including reduced weight loss, decreased DAI score, relief of colon shortening and lower infiltration of inflammatory cell in the colonic mucosa (Fig. 2B–F). Additionally, significant reductions were observed in markers of both systemic and colonic inflammation when administered with phenolic acids (Supplementary Fig. 3A-F). In line with DSS colitis model results, the secretion of TNF-α, IL-1β, and IL-6, were also obviously reduced in response to LPS when administered with phenolic acids (Supplementary Fig. 3G).

Since more inflammatory cells infiltration and elevated levels of TNF-α, IL-1β, and IL-6 were observed in colonic tissue of DSS-treated mice, we next examined changes of cellular components involved in the colitis with a special focus on macrophages. To verified whether phenolic acids-mediated reduction of colitis was achieved through the modulation of macrophage polarization, we analyzed the expression of M1 macrophage signature genes and the frequency of CD11b + CD11c+, M1 macrophages in the colon of mice. As expected, CGA and FA a significant decrease in the frequency of CD11b + CD11c+, M1 macrophages in the colon (Fig. 2G, H). RT-PCR also confirmed that the elevated mRNA levels of *Nos-2* and *Cxcl9* were down-regulated in the presence of CGA or FA (Fig. 2I). The analysis of macrophage phenotype revealed a notable reduction in pro-inflammatory M1 macrophage. To further investigate the impact of phenolic acid on macrophage polarization, we assessed the characteristics of LPS-stimulated murine RAW264.7 cells, both in the presence and absence of phenolic acid. The mRNA expression of M1-characterized *Il-1β*, *Il-6*, *Tnf-a*, *Nos-2*, and *Cxcl9* were also significantly reduced in response to LPS when pre-treated with CGA and FA, but not CA or EA (Supplementary Fig. 3H). These data suggest that phenolic acid reduced intestinal inflammation potentially inhibiting macrophage polarization to the M1 phenotype.

### Chlorogenic acid modulates colitis severity through the macrophage-NLRP3 axis

**Fig. 3:**
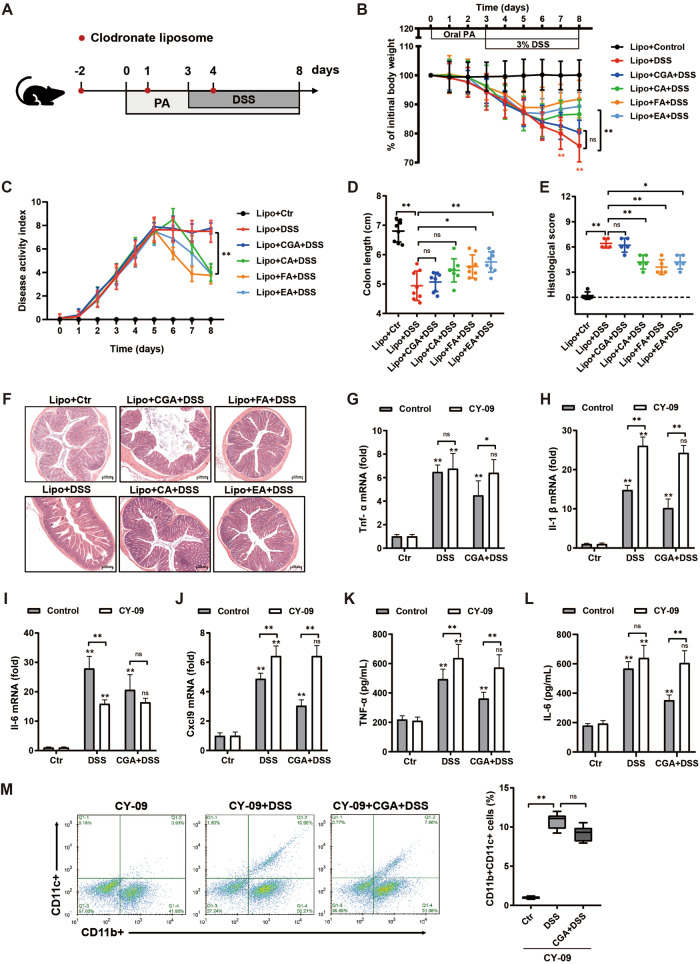
Chlorogenic acid fails to ameliorate colitis in either macrophage-depleted or Nlrp3-deficicent mice. **A** Schematic outline of the experimental design. **B**–**D** Colitis severity was measured as body weight loss, DAI, and colon length (*n* = 8). **E, F** H&E staining of colon tissue and histological scores at day 8 after oral phenolic acids. **G**–**J** The mRNA expression of M1-characterized *Tnf-a*, *Il-1β*, *Il-6*, and *Cxcl9* in the colon of CY-09-treated mice and control littermates. **K, L** Secretion of TNF-α and IL-6 in serum from CY-09-treated mice and control littermates. **M** Flow cytometry analysis of pro-inflammatory CD11b + CD11c+ colonic macrophage (*n* = 6). Data represent means ± SD of three separate experiments. Statistics was performed with unpaired two- sided Student’s *t* test or one-way ANOVA, followed by Tukey’s multiple comparison test. *P < 0.05, **P < 0.01.

To study if the protective effects of phenolic acids on colitis was dependent upon macrophage, we depleted macrophage by in vivo administration of clodronate-loaded liposomes on 2 days before phenolic acid treatment, and also on day 1 and day 4 during phenolic acid treatment, and on day 3, acute experimental colitis was induced with 3% DSS in drinking water given ad libitum for 5 days, as illustrated in Fig. 3A. However, the colitis symptoms were notably alleviated by caffeic acid, ferulic acid and ellagic acid as both histological and serological markers of inflammation were also markedly abrogated (Fig. 3B–F). Notably, the effectiveness of chlorogenic acid in reducing colitis severity was not observed in mice with depleted macrophages (Fig. 3B–F), indicating the necessity of macrophages for the protective effects mediated by chlorogenic acid. The M1 macrophage phenotype is characterized by the activation of Nlrp3 and the induction of proinflammatory mediators such as IL-1β, IL-6, TNF-α, and CXCL9 [36, 37]. Hence, we next sought to determine whether Nlrp3 can be targeted to treat the severe colitis by regulating macrophage polarization. CGA abolished the increased expression of either M1 signature cytokines or gene *Nlrp3* in colonic tissue of DSS-treated mice (Fig. 3G–J, and Supplementary Fig. 4A). To further confirm the dependence of chlorogenic acid’s alleviation of DSS-induced colitis on the intervention of the Nlrp3 inflammasome, we verified in CY-09-, an inhibitor of Nlrp3, treated mice. The NLRP3 mRNA level of CY-09-treated mice was significantly decreased relative to that of controls (Supplementary Fig. 4B). As expected, CGA could not reduce the markers of systemic inflammation caused by DSS in CY-09-treated mice, while CY-09-treated mice showed more higher gene expression of inflammatory factors than control mice (Fig. 3K, L). Next, we investigated if deletion of Nlrp3 might reverse the effect of CGA on M1 polarization. Here, we observed that CGA did not improve DSS-induced the induction of pro-inflammatory M1-macrophage in CY-09-treated mice because the transcripts of *Tnf-α*, *Il-1β*, *Il-6*, and *Cxcl9* were significantly increased (Fig. 3G–J). Similarly, flow cytometry analysis revealed the percentage of CD11b + CD11c+ M1-macrophage was not decreased by CGA in colonic tissue of Nlrp3-deficient mice during colitis (Fig. 3M). These data indicate that the critical role of Nlrp3 in the protection conferred by CGA against DSS-induced colitis.

Given that CGA did not modulate colitis severity in either macrophage-deleted or CY-09-treated mice, we next considered the possibility that the protective effect of CGA against colitis might be achieved by macrophage-NLRP3 axis. To understand how Nlrp3-deleted macrophages influence intestinal inflammation, LPS-stimulated Caco2 cells were co-cultured with either Nlrp3-WT or Nlrp3-KD macrophages, respectively (Supplementary Fig. 4D). The efficiency of Nlrp3 siRNA was confirmed by RT-PCR (Supplementary Fig. 4C). As expected, LPS significantly reduced TEER of Caco2 cells, while increasing inflammatory cytokine levels in co-culture system. Further, depletion of Nlrp3 in macrophages exacerbated this IEC barrier defect, as evidenced by a marked decrease of TEER and higher secretion of cytokines, consistent with reduced intestinal barrier integrity in Caco2 cells. Intriguingly, CGA reversed these barrier-disrupting effects that was observed in Caco2 cultured with Nlrp3-WT, but not Nlrp3-KD, macrophages (Fig. 3E–G). Collectively, these data confirm that loss of Nlrp3 in macrophages compromises intestinal epithelial barrier function demonstrating that CGA exhibited the ability to mitigate epithelial inflammation and enhance intestinal barrier function depending on macrophage-Nlrp3 axis.

### Anti-inflammatory effect of chlorogenic acid is mediated through PKM2-dependent glycolysis

**Fig. 4:**
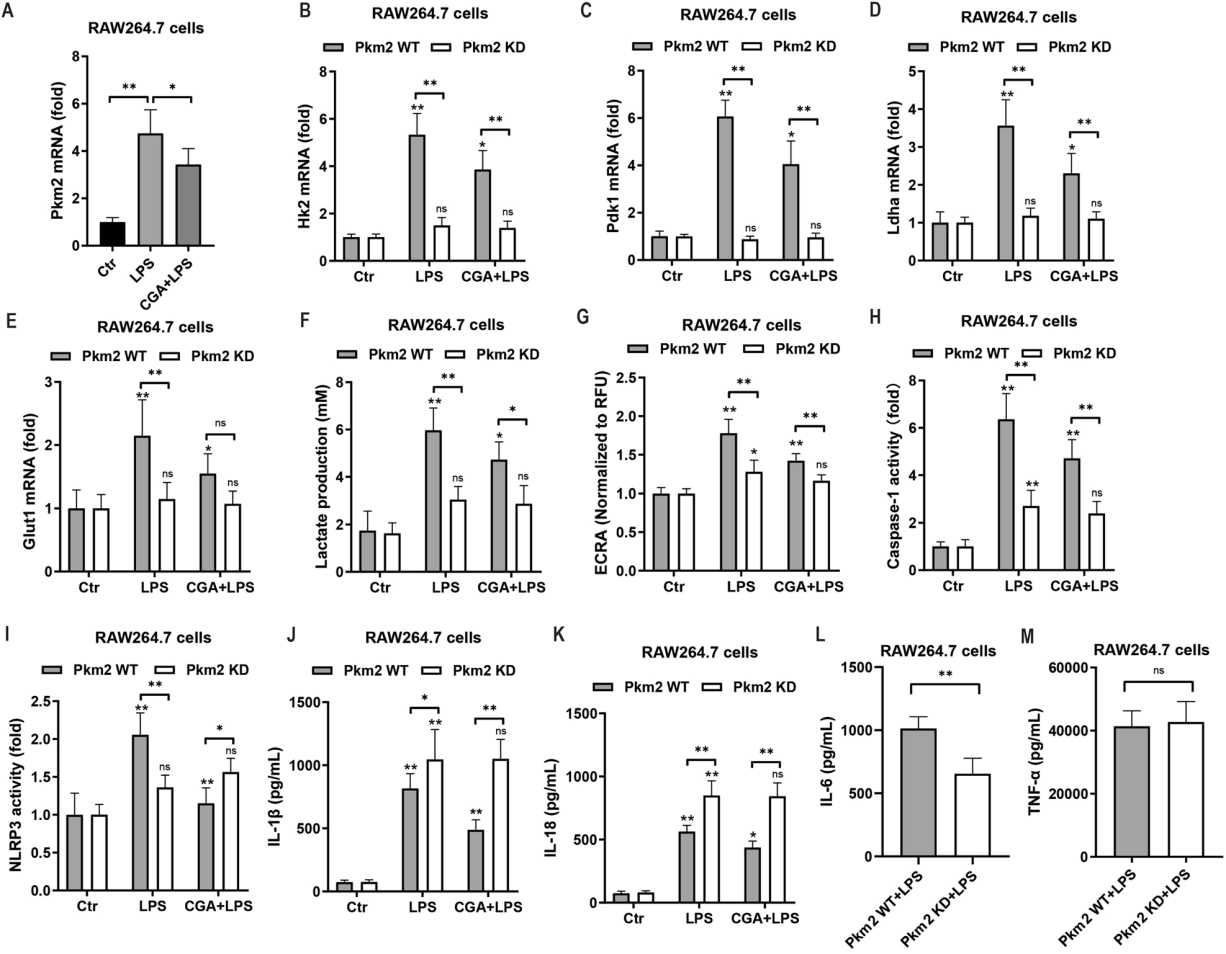
Chlorogenic acid exerts anti-inflammatory effect by reducing PKM2-depdent glycolysis of macrophage. **A**–**E** RT-qPCR analysis of *Pkm2*, *Hk2*, *Pdk1*, *Ldha*, and *Glut1* mRNA levels. **F, H, I** ELISA of lactate production, caspase-1 and NLRP3 activity. **G** The Seahorse XF-96 Extracellular Flux Analyzer of ECRA. **J**–**M** Secretion of IL-1β, IL-18, IL-6 and TNF-α in RAW264.7 cells (*n* = 6). Data represent means ± SD of three separate experiments. Statistics was performed with unpaired two- sided Student’s *t* test or one-way ANOVA, followed by Tukey’s multiple comparison test. *P < 0.05, **P < 0.01.

Next, we sought to gain a better mechanism understanding of how CGA inhibits the activation of Nlrp3 and consequently diminishes intestinal inflammation. Recent studies indicated that there is a link between inflammatory and glycolytic responses, specifically, the regulation of Nlrp3 priming and activation by increased glycolysis [38]. Therefore, we aimed to investigate whether CGA has any modulatory effect on glycolysis of macrophage, and eventually on Nlrp3 activation in response to LPS. Challenge with LPS highly induced the mRNA expression related to glycolysis, such as pyruvate kinase M 2 (*Pkm2*), hexokinase 2 (*Hk2*), pyruvate dehydrogenase kinase 1 (*Pdk1*) and lactate dehydrogenase A (*Ldha*), while CGA significantly decreased this up-regulated genes level in macrophages (Fig. 4A–D). We also observed a significant reduction in the LPS-induced increased glucose transporter protein 1 (*Glut1*) mRNA expression and augmented lactate production in the presence of CGA (Fig. 4E, F). Importantly, the enhanced extracellular acidification rates (ECRA), indicative of glycolysis, was rescued by CGA treatment (Fig. 4G). Furthermore, CGA had no effect on cell viability (Supplementary Fig. 5A). This indicated that CGA may exert anti-inflammatory effect by mediating glycolysis.

To examined if CGA-mediated protection was associated with the metabolic phenotype of macrophages, we subsequently targeted PKM2, the final rate-limiting reaction in the glycolytic pathway. Knockdown of *Pkm2* by RNAi led to reduced mRNA expression of *Hk2*, *Pdk1*, *Ldha*, and *Glut1* in macrophage stimulated with LPS (Fig. 4B–E, and Supplementary Fig. 5B). In addition, inhibition of PKM2 significantly prevent increases in lactate level, the extracellular acidification rates, as well as caspase-1 and Nlrp3 activation, IL-1β, IL-18 and IL-6 but not TNF-α release by macrophages following treatment with LPS (Fig. 4F–M). However, CGA significantly inhibited LPS-induced increases in gene expression of *Hk2*, *Pdk1*, *Ldha*, and *Glut1* in PKM2-WT but not PKM2-KD macrophages (Fig. 4B–E). As a consequence, the inhibitory effect of CGA on ECRA and lactate production was rescued by PKM2 deletion (Fig. 4F, G), showing that inhibition of PKM2 mediated the downregulation of glycolysis by CGA. Furthermore, the decrease in the activation of capase-1 and Nlrp3, as well as the secretion of IL-1β and IL-18 caused by CGA treatment in LPS-induced macrophages could not be attenuated by knockdown of PKM2 (Fig. 4G–K). Collectively, these findings providing evidence that the anti-inflammatory effect of CGA can be overcome by reducing PKM-dependent glycolysis. We next ask whether the glycolytic changes described above in PKM2-deficient macrophages resulted from mitochondrial dysfunction. CGA exhibited the capability to mitigate dysfunctional mitochondria characterized by diminished membrane potential and a high level of MitoSOX, while this protective effect was attenuated in PKM2-deficient macrophages (Supplementary Fig. 5C, D). All above data effectively demonstrate that CGA exerts anti-inflammatory effect by inhibiting PKM2-dependent glycolysis and maintaining mitochondrial function, and thereby modulating macrophage-Nlrp3 axis to prevent M1 polarization.

### Ferulic acid alleviates colitis by suppressing the formation of neutrophil extracellular traps

**Fig. 5:**
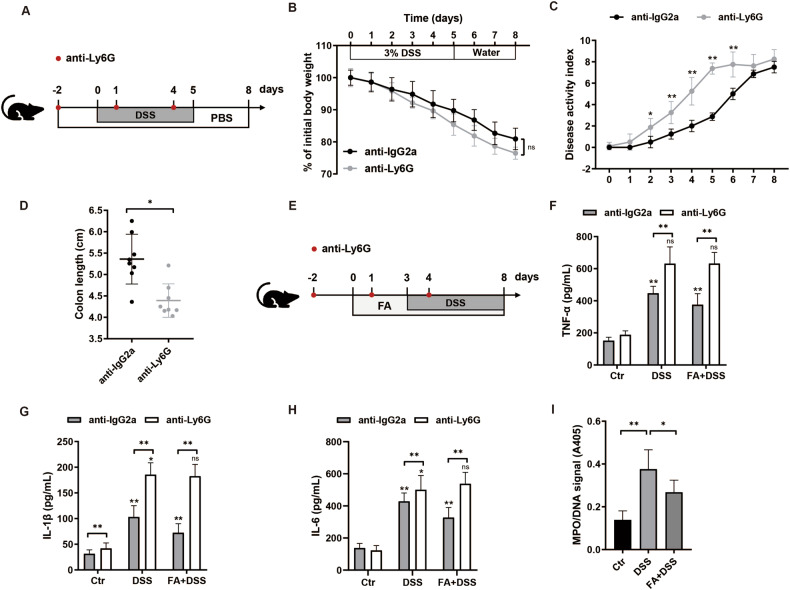
Ferulic acid ameliorates DSS-induced colitis at least partially through inhibition of neutrophil extracellular traps formation. **A** Mice were intraperitoneally injected with anti-IgG2a or anti-Ly6G on 2 days before 3% DSS treatment, and also on day 1 and day 4 during DSS treatment. **B**–**D** Colitis severity was measured as body weight loss, DAI, and colon length (*n* = 8). **E** Schematic outline of the experimental design. **F**–**H** Secretion of TNF-α, IL-1β and IL-6 in serum. **I** ELISA of the MPO-DNA signals (*n* = 6). Data represent means ± SD of three separate experiments. Statistics were performed with unpaired two-sided Student’s *t* test or one-way ANOVA, followed by Tukey’s multiple comparison test. *P < 0.05, **P < 0.01.

Morphologically, FA displayed the ability to induce less infiltration of inflammatory cells in the colonic mucosa (Fig. 2E). Flow cytometric analysis revealed a significant reduction in the colonic infiltration of neutrophils in mice treated with FA compared to those in the vehicle control group (Supplementary Fig. 6A). Furthermore, administration of FA led to a decrease in the production of *Il-17* and *Il-22* by neutrophils (Supplementary Fig. 6B). We then directly evaluated the role of neutrophils during colitis by in vivo administration of specific Ly6G depleting antibody on 2 days before the initiation of 3% DSS treatment and subsequently on day 1 and day 4 during the DSS treatment period (Fig. 5A). Based on flow cytometry, the Ly6G positive cells were significantly decreased in anti-Ly6G-injected mice (60.27 ± 2.24%) compared to 26.25 ± 1.70% in the control group (Supplementary Fig. 7). Mice lacking neutrophils were treated as recipients in colitis model in the presence or absence of FA supplements (Fig. 5E). Depletion of neutrophils significantly extended the colitis symptoms, as evidenced by the increased disease activity indices and colon shortening, but not the percentage of weight loss (Fig. 5B–D). To determine the mechanism by which neutrophils contribute to colitis, we examined the production of inflammatory factors including TNF-α, IL-1β, and IL-6. The secretion of TNF-α and IL-1β, but not IL-6 were profoundly reduced in the colon of neutrophil-depleted mice after DSS treatment. However, FA did not abrogate the elevated inflammatory cytokines in neutrophils-deleted mice (Fig. 5F–H), showing that neutrophils contribute to the resolution of inflammation mediated by FA. In exploring whether FA could prevent colitis development by regulating neutrophil functions, we postulated that FA-mediated protective effect might be attributable to the formation of neutrophil extracellular traps (NETs). The NET formation was confirmed by quantification of DNA-bound MPO. FA treatment significantly decreased the MPO/DNA signals in neutrophils from DSS colitis mice (Fig. 5I), indicating that FA mitigates DSS-induced colitis, at least in part, through inhibition of NETs formation.

### The beneficial effect of caffeic acid and ellagic acid is microbiota-dependent

**Fig. 6:**
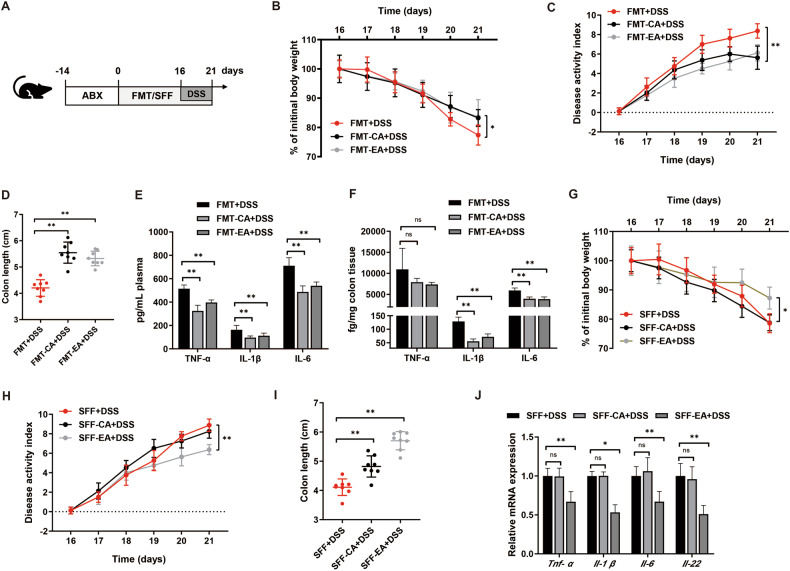
Caffeic acid and ellagic acid alleviate DSS-induced colitis in a microbiota-dependent manner. **A** Schematic outline of the experimental design. **B**–**D** Colitis severity was measured as body weight loss, DAI, and colon length (*n* = 8). **E, F** Secretion of TNF-α, IL-1β and IL-6 in serum and colon homogenates of mice receiving fecal microbiota from CA or EA-administrated donors. Gut microbial metabolites from ellagic acid transferred into DSS model could reduce colitis. **G**–**I** Colitis severity was measured as body weight loss, DAI, and colon length (*n* = 8). **J** RT-qPCR analysis of *Tnf-α*, *Il-1β*, *Il-6* and *Il-22* mRNA levels in colon homogenates of mice receiving microbial metabolites from CA or EA-administrated donors. Data represent means ± SD of three separate experiments. Statistics was performed with one-way ANOVA, followed by Tukey’s multiple comparison test. *P < 0.05, **P < 0.01.

In order to investigate whether the protective effect of phenolic acids on disease severity relied on the gut microbiota, wild-type mice were exposed to a broad-spectrum antibiotic cocktail to perturb gut microbiota before DSS treatment (Fig. 6A). We found that, relative to the DSS colitis model, both CA and EA could ameliorate the systemic and intestinal inflammation (Fig. 2). However, supplementation of the microbiota-depleted mice with CA or EA did not prevent colitis severity, demonstrating that the influence of CA and EA in colitis severity is microbiota-dependent (Supplementary Fig. 8). To assess whether the altered microbiota resulting from CA or EA consumption could individually contribute to reduced severity in the colitis model, we performed fecal microbiota transplantation (FMT) by transferring feces from mice that had consumed either CA or EA into DSS-treated recipients. However, following transplantation, no significant difference in colitis severity was observed among the recipients of the microbiota transplants. Mice receiving fecal microbiota from CA or EA-administrated donors exhibited the reduced percentage of weight loss, decreased disease activity indices, and colon shortening (Fig. 6B–D). Besides, FMT rescued the elevated inflammatory factors TNF-α, IL-6, and IL-1β in serum and colonic tissue of mice colitis model (Fig. 6E, F), indicating that both of CA- and EA-altered microbiota can effectively transfer the colitis protection.

Given the pivotal effect of gut microbes and microbiota-derived signals in control inflammation [19, 20], we therefore transplanted the metabolites of fecal microbiota obtained from mice subjected to either CA or EA gavage to DSS-treated mice. Intriguing, when the metabolites produced by the gut microbiota from the EA group were transferred to colitis mice, there was a significant reduction in weight loss, DAI, colon shortening, and macroscopic colitis scores, in comparison to the colitis controls. (Fig. 6G–I). Further, the mRNA levels of *Tnf-α*, *Il-1β*, *Il-6,* and *Il-22* were also markedly decreased in the colons of metabolites transplant recipients (Fig. 6J). Dissimilarly, we observed that, relative to colitis model, no significant difference in colitis severity was observed in the mice receiving the metabolites produced by CA in the presence of gut microbes (Fig. 6G–J). Metabolite transplant experiments suggest that intestinal microbial metabolites from EA-treated, but not CA-administrated, mice displayed an anti-inflammatory effect, showing that the protective effect of EA was achieved by the alternations of intestinal microbial metabolites.

### Urolithin A mitigates colitis and enhances gut barrier function in an IL22-dependent manner

**Fig. 7:**
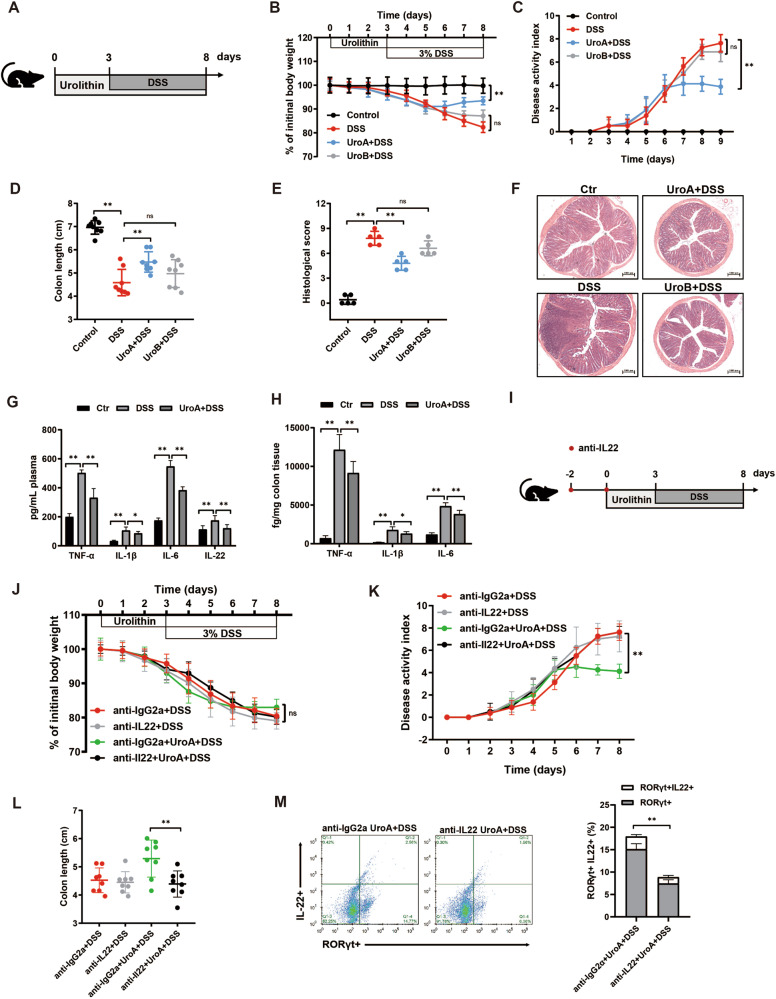
IL-22 is essential for the protective effects of urolithin A against colitis. **A** Schematic outline of the experimental design. **B**–**D** Colitis severity was measured as body weight loss, DAI, and colon length (*n* = 8). **E, F** H&E staining of colon tissue and histological scores at day 8 after oral urolithin. **G, H** Secretion of TNF-α, IL-1β, IL-6 and IL-22 in serum, and the levels of TNF-α, IL-1β and IL-6 in colon homogenates. **I** Mice were intraperitoneally injected with anti-IgG2a or anti-IL22, and urolithin A was given to mice by oral gavage on day 3 of the DSS colitis model. **J**–**L** Disease severity was measured as body weight loss, DAI, and colon length (*n* = 8). **M** Flow cytometry of RORγ + IL22+ innate lymophoid cell populations from urolithin A-administrated mice at day 8 after DSS challenge. Data represent means ± SD of three separate experiments. Statistics was performed with unpaired two- sided Student’s *t* test or one-way ANOVA, followed by Tukey’s multiple comparison test. *P < 0.05, **P < 0.01.

Overall, previous studies have shown that ellagic acid and its metabolite urolithin contributed to the prevention against the detrimental effect of inflammation [29]. Based on this information, urolithin supplementation experiments were performed instead of transferring microbiota and their metabolites to determine what EA effects, if any, could potentially be attributed to an increase in urolithin production. In this context, DSS colitis mice were treated with UroA and UroB, as outlined in Fig. 7A. Notably, when replacing EA with UroA, similar results and outcomes were observed. UroA, but not UroB treatment, led to less severe colitis, as exemplified by the reduced weight loss, DAI, colon shortening, and macroscopic colitis scores compared to the colitis control group (Fig. 7B–F). Moreover, UroA treatment suppressed the secretion of inflammatory cytokines IL-1β, TNF-α, IL-6, and IL-22 (Fig. 7G, H). Given the proximity of microbial metabolites to the gut epithelium, it was suggested that UroA might directly influence the function of epithelial cells. In vitro, characteristics of LPS-stimulated Caco2 cells in the presence or absence of UroA were assessed. As expected, UroA treatment relieved the barrier defects, as preserved the reduced TEER and increased tight junction-related gene expression of Occludin, Claudin-4, and ZO-1 have been observed (Supplementary Fig. 9A, B). These observations indicated that UroA was able to mitigate colitis and promote gut barrier integrity.

To gain deeper insights into the mechanism underlying the enhanced barrier function, we next determine if the secretion of IL-22 was necessary for the protective effects of UroA during colitis. UroA treatment of DSS colitis experiments was repeated to include a neutralizing antibody against IL-22 (Fig. 7I). Based on flow cytometry, the IL22 positive cells were significantly decreased in anti-IL22-injected mice (18.50 ± 3.66%) compared to 6.75 ± 1.99% in the control group (Supplementary Fig. 10A). Interestingly, UroA treatment with the neutralizing antibody failed to prevent colitis-associated weight loss, DAI, colon shortening nor damage to the colon (Fig. 7J–L). More importantly, we found the loss in the ability of UroA to induce the reduction of innate lymphoid cells (ILC3s) number after IL-22 neutralization during colitis (Fig. 7M, and Supplementary Fig. 10B). The results raised the possibility that IL-22 was responsible for the protective effects of UroA against colitis.

## Discussion

This study aims to elucidate whether the gut microbiota and macrophage involves in the impact of phenolic acids on intestinal inflammation. Towards this, we used macrophage-deleted or microbiota- mice administrated with four unique phenolic acids in DSS models of colitis to identify how dietary polyphenol influences the interactions between macrophage and microbiota that modulate colitis severity. Our results supported that phenolic acid protects against colitis in various distinct manners. Specifically, chlorogenic acid alleviated colitis in a macrophage-dependent mechanism. However, ferulic acid-mediated reduction of colitis was achieved at least partially in a neutrophil-dependent manner. Furthermore, caffeic acid and ellagic acid exerts anti-inflammatory effect that is dependent upon gut microbiota. IL-22 were responsible for the mitigative effects of urolithin A, the metabolite produced by ellagic acid in the presence of gut microbes, on gut barrier integrity and inflammation.

The plasticity of macrophages could enable them to react to a changing microenvironmental cues, which direct the polarization of macrophages towards to distinct phenotypes including pro-inflammatory (M1) and anti-inflammatory (M2) macrophages [9]. A balance in M1/M2 macrophage polarization is believed critical to the inflammation resolution and intestinal homeostasis [4, 8]. Hence, therapeutic approaches focusing on macrophage polarization and function by either re-programming macrophage polarization, or skewing M1 macrophage toward the M2 phenotype present potential clinical promise in IBD [10, 39]. Dietary-derived polyphenol was able to promote the phenotypic conversion of M1 to M2 macrophages in the alleviation of colitis [40]. Correspondingly, we demonstrated a requirement of macrophages in the chlorogenic acid-mediated protection. NLRP3 inflammasome is critically important in reprogramming M1 macrophage polarization and inducing IL-1β production [37]. NLRP3 is required for chlorogenic acid amelioration of colitis, whereas knockdown of NLRP3 undermined the reversed effects of chlorogenic acid. Given the cellular metabolic profiles of macrophages is key components of macrophage polarization, instrumental to their function in resolving inflammation resolution [11, 12], the data presented here indicate that anti-inflammatory effect of CGA can be overcome by reducing PKM-dependent glycolysis. PKM2 deficiency reduced IL-1β release through inhibition of NLRP3 inflammasome. In accordance with a recent study, our findings demonstrated that glycolysis driven by PKM2 contributes to the regulation of IL-1β release by activating the NLRP3 inflammasome [41, 42]. We provide the first evidence that chlorogenic acid mitigate colitis and prevent colitis-caused dysbiosis through inhibiting PKM2-dependent glycolysis, and thereby blocking Nlrp3 activation to shift the inflammatory M1 macrophage toward the anti-inflammatory M2 phenotype. Even though ferulic acid was capable of prevent M1 macrophage polarization, the elevated colonic markers of inflammation were also abrogated by ferulic acid in macrophage-deficient mice. The coordinated interaction between neutrophils and distinct subsets of macrophage are vital for tissue repair processes [43, 44]. Neutrophils were recently demonstrated to involved in triggering macrophage polarization towards a reparative phenotype [45], as well as priming macrophages for transcription of IL-1β by releasing NETs [46]. Considering the neutrophil-macrophage cooperation and its impact on inflammation resolution, we found that for the first time ferulic acid amelioration of colitis is neutrophil-dependent and achieved partly through blocking the formation of NETs, in line with recent investigations indicating that in vivo inhibition of NETs release attenuated colitis [47].

Accumulating evidence confirmed that the beneficial effect of phenolic acid in maintaining gut health was overcome through altering gut microbes [25, 30, 31]. We conducted to clarify the causative link between phenolic acids’ modulation of microbiota and colitis alleviation effects. Neither caffeic acid nor ellagic acid modulates colitis severity in microbiota-depleted and DSS-induced mice, supporting a central role of the polyphenol-gut microbiota interplay in alleviating intestinal inflammation. These findings improve our understanding of the causality of phenolic acid-altered gut microbes and their metabolic profiles in anti-colitis activities and will serve as a guiding principle for the future development of therapeutic approaches targeting IBD. To further understand the role of the gut microbiota and their associated metabolic products in polyphenol-mediated health benefits, we employed FMT to explore how modulated gut microbiota might impact disease prevention through polyphenol administration. Previously, in the DSS-induced colitis model, colitis-induced mice receiving fecal materials from green tea polyphenol-fed donors, rather than those from controls, ameliorated colitis symptoms [26]. Herein, we reported that transferred fecal microbiotas from either caffeic acid- or ellagic acid-administrated mice to colitis recipients could attenuate colitis, which in turn helps explain why gut microbes are indispensable in caffeic acid- and ellagic acid-mediated protection of mice from DSS colitis. It is increasingly recognized that metabolites produced by gut microbes, as a central regulator of inflammation, trigger inflammatory responses and correlate with inflammatory-related diseases [8, 47, 48]. The metabolites generated via gut microbiota-mediated catabolism of polyphenol exhibited anti-inflammatory benefits, promote gut barrier integrity and therefore play significant roles in countering colitis [18, 29, 49]. Our data in mice receiving metabolites produced by bacteria from dietary ellagic acid, rather than caffeic acid, show reduced colitis and enhanced gut barrier function. These findings were important in understanding bacterially derived metabolites serve as important signals, shedding lights on alleviating colitis by ensuring the effective functioning of the epithelial barrier. It was confirmed that gut microbes were able to metabolize ellagic acid to urolithin A and urolithin B. Our current findings in agree with a previous study showing that direct administration with urolithin A, but not urolithin B, offers health benefits evidenced by increased gut barrier protection and anti-inflammatory activities [29]. Nevertheless, it is entirely unknown how urolithin A mitigates the host’s intestinal inflammation. In this regard, we also demonstrated a requirement of IL-22 in urolithin A-mediated gut barrier protection. Consistently, a recent report showed intestinal microbiota-derived metabolites enhances immune cell IL-22 production that protects intestine from inflammation [50].

Overall, our results demonstrated that phenolic acid protects against DSS-induced colitis in various distinct manners. Specifically, chlorogenic acid alleviated colitis through reducing PKM2-dependent glycolysis and inhibiting NLRP3 activation that shift macrophage polarization. Rather, ferulic acid reduces colitis at least partially through inhibition of NETs formation. Caffeic acid and ellagic acid modulate colitis severity dependence on gut microbiota. Gut microbial metabolites from ellagic acid transferred into DSS model could reduce colitis. Especially, urolithin A, a metabolite produced by ellagic acid in the presence of gut microbes, mitigates colitis in IL-22-dependent manner. The oral phenolic acids administration in animals improves the gut barrier integrity and reduces intestinal inflammation from the interaction between macrophage-neutrophil and gut microbiota in various distinct manners, including the reduction of pro-inflammatory molecules associated with the skewing macrophage polarization and inhibition of NETs formation, the improvement in tight-junction protein expression related to the composition of gut microbiome that favoring an increase in beneficial bacteria and their metabolites, suggesting the potential use of phenolic acids in the management of intestinal inflammation development. However, further investigations are needed to determine whether the protective effects of phenolic acids are responsible in IBD patients, as well as whether phenolic acids could influence the interaction of gut microbiota with macrophage or whether other factors are involved in the beneficial effects of phenolic acids against intestinal inflammation. Moreover, further studies are needed to explore the underlying mechanisms of phenolic acids in the mediation of protection against intestinal inflammation (e.g., the receptor of substances the downstream signals).

## Material and methods

### Mice

For animal studies, male wild-type C57BL/6 mice were kept in specific pathogen-free (SPF) conditions with free access to a standard chow diet and drinking water. All mouse procedures were approved by the China Agricultural University Animal Care and Use Committee under protocol number AW51211202-1-3.

### DSS-induced colitis

To induce acute experimental colitis, 3% dextran sulfate sodium (DSS; 36-50 kDa; MP Biomedicals, CA, USA) was provided in drinking water ad libitum for 5 days, then followed with regular water for another 3 days. Colitis severity was evaluated by monitoring the changes of body weight, histological scores, DAI, and colon length. The entire colon of each mouse was removed, and the colon length was recorded.

### Antibiotic treatment and fecal transfer studies

To deplete intestinal microbiota, four-week-old mice were selected for one week adaptation period and then given four-antibiotic (ABX) cocktail in the drinking water containing 1 g/L streptomycin, 0.5 g/L ampicillin, 1 g/L gentamicin, and 0.5 g/L vancomycin for 2 weeks. The efficiency of bacterial depletion was confirmed as described [51]. A five-fold sterile PBS containing 15% glycerol (v/v) was used to dilute fresh feces collected from indicated mice. Fecal samples were resuspended, and then filtered by sterile gauze and 0.22 μm filters to obtain the fecal microbial suspension and sterile fecal filtrate, respectively.

For phenolic acid intervention study, mice aged 7 weeks were gavaged once daily with 100 μL PBS containing 50 mg/kg body weight of CA and EA for two weeks. For fecal transfer experiments, mice were orally administrated with 200 μL of fecal microbial suspension and sterile fecal filtrate from individual donors receiving either PBS or CA- and EA-treated mice once every other day for indicated times.

### Macrophage/neutrophil depletion and phenolic acid treatment

For macrophage depletion, mice were intraperitoneally injected of 200 μL clodronate liposomes (Amsterdam, The Netherlands) once every 3 days for three times [52]. For neutrophil depletion, mice were administrated intraperitoneally with 200 μg anti-Ly6G (clone 1A8, BD Biosciences), isotype control IgG2a antibodies (clone RTK2758; BioLegend), or phosphate-buffered saline (PBS) once every 3 days for three [45]. For phenolic acid treatment, mice were intraperitoneally injected of either 200 μL clodronate liposomes or 200 μg anti-Ly6G once every 3 days for three times on 2 days before phenolic acid treatment with 50 mg/kg body weight, as well as on day 1 and day 4 during treatment with phenolic acid.

### Bone marrow-derived macrophage (BMDM) isolation and differentiation

Six-week-old C57BL/6 mice were flushed of BMDMs from the femur and tibia. The isolated BMDMs were differentiated in RPMI-1640 medium supplemented with 10% FBS and 20 ng/mL macrophage-colony stimulating factor (MCSF) for 5 days. On day 5 during DSS treatment, macrophage-depleted mice were given by intraperitoneal injection 5 × 10^5^ BMDMs in 100 μL PBS per mouse.

### Il-22 neutralization

For IL-22 neutralization, an anti-IL22-specific mAb (50 μg per mouse; clone 2G12A41, BioLegend) was intraperitoneally injected twice every 2 days to mice, with isotype control IgG2α mAb serving as the control groups [53].

### Histology

The colons of mice were harvested and stained with hematoxylin and eosin (H&E) and immunofluorescence, respectively, in accordance with standard protocols. For histological analysis, the histology score was determined as previously described.

### Flow cytometry

A segment of the colon tissue was minced and incubated in magnesium and calcium-free Hank’s Balanced Salt Solution containing 5 mM ethylenediaminetetraacetic acid and 1 mM dithiothreitol at 37 °C for 30 min with shaking. Then, colon pieces were digested with RPMI-1640 medium containing 0.05% collagenase D, 0.05% DNase I, and 2% new-born calf serum for 45 min with gentle shaking. Single cells were obtained by filtering through 100-µm nylon-mesh cell strainers. After centrifugation for 5 min at 12,000 rpm, the cells were harvested and then incubated with anti-CD11b (clone M1/70, BioLegend), anti-CD11 (clone N418, BioLegend), anti-Gr-1 (clone RB6-8C5, BioLegend), anti-Ly6G (clone 1A8, BioLegend), anti-RORγt (clone 4F3-3C8-2B7, BioLegend), anti-IL22 (clone 2G12A41, BioLegend) for 30 min. Following three washing, cells were sorted by flow cytometry using the BD FACS Aria II Cell Sorter (BD Biosciences, New Jersey, USA). Acquired data were gated and analyzed with FlowJo software.

### Cell culture and co-culture

Intestinal epithelial Caco2 cells, murine macrophage RAW264.7 cells, and BMDMs were cultured in complete DMEM supplemented with 10% (v/v) heat-inactivated FBS at 37 °C with 5% CO_2_. For co-culture system, IECs grown on permeable transwell inserts were co-cultured with RAW264.7 cells seeded in 12-well plates for indicated time before collecting the supernatants for subsequent assays as previously described [54].

### RNA interference and RT-PCR

After transfection, the extraction of total RNA was performed using the TRIzol reagent (TransGen, Beijing, China), followed by the conversion of the RNA into cDNA using the iScript first-strand cDNA synthesis kit (TransGen, Beijing, China). For real-time quantitative PCR, SYBR Green was utilized on an ABI ViiATM 7 Real-Time System (Thermo Fisher Scientific, USA). The normalization of threshold cycle numbers was achieved using the housekeeping genes *β-actin* and *Gapdh*. Primer sequences are listed in Table S1.

### Cell viability assay

Cell viability was assessed using Cell Counting Kit-8 (CCK-8) solutions from Dojingdo Laboratories (Kumamoto, Japan), following the guidelines provided by the manufacturer. The absorbance at 450 nm was then measured using a microplate reader from Bio-Rad (CA, USA).

### Mitochondrial member potential and MitoSOX assay

The assessment of mitochondrial membrane potential and superoxide in RAW264.7 cells were performed using Mitochondrial Membrane Potential Assay Kit (Abcam, Cambridge, USA), and cell permeable dye MitoSOX (Thermo Fisher Scientific Inc., USA), respectively, as recommended by each manufacturer. Signal intensity analysis was conducted using a fluorescent microplate reader (Tecan, Mannedorf, Switzerland).

### Lactate production

The lactate concentration in cell supernatants was quantified using the Lactate Assay Kit from ML BIO Biotechnology Co., Ltd (Shanghai, China), following the instructions provided by the manufacturer.

### Glycolysis assay

Cellular glycolysis in RAW264.7 cells was evaluated using the Seahorse XF-96 Extracellular Flux Analyzer (Seahorse Bioscience, USA), as described [55]. Following trypsinization, a total of 10^4^ cells were seeded into the wells of Seahorse XF-96 plates with 80 μL of growth media, followed by an overnight incubation period. Subsequently, to assess extracellular acidification rates (indicative of glycolysis), a mixture containing 10 mM glucose, 0.5 μM oligomycin, and 100 mM 2-deoxy-glucose was introduced, following the manufacturer’s protocols.

### Caspase-1 and NLRP3 activation

The Caspase-1 and NLRP3 activation in colonic and cellular lysis was measured using Caspase-1 Assay Kit (ab39412, Abcam, Cambridge, MA, USA) and NLRP3 ELISA Kit (ab279417, Abcam, Cambridge, MA, USA), respectively, following the instructions provided by the manufacturer.

### Cytokine measurements

The concentrations of TNF-α, IL-1β, IL-6, IL-10, IL-18, and IL-22 in mouse serum, colonic tissue homogenates, and cell culture supernatants were measured using commercial enzyme-linked immunosorbent assay (ELISA) kits from BD Biosciences. The quantification was carried out following the instructions provided by the respective manufacturers.

### MPO-DNA complex (NET) ELISA

Primary neutrophils were isolated from peripheral blood as described [56]. To detect and quantify soluble NETs in cell lysis supernatants, the MPO-DNA complex ELISA method was employed, following the instructions provided by the manufacturer. In brief, 96-well ELISA Maxisorp plates (Thermo Fisher) were coated with mouse anti-mice MPO antibody at a concentration of 5 mg/mL and subsequently blocked using 1% BSA. After washing three times, a mixture of 80 μL of incubation buffer, 20 μL of tissue lysis supernatant, and 4 μL of peroxidase-labeled anti-DNA monoclonal antibody was added to each well for a 2 h incubation. Following a single wash, 100 μL of peroxidase substrate was added. After a 20 min incubation, the absorbance was measured at 405 nm using a microplate reader from Bio-Rad (CA, USA).

### Statistical analyses

Quantitative data were shown as means ± standard deviation. Statistical analyses were conducted with two-tailed, Student’s *t* test or one-way analysis of variance (ANOVA), followed by Tukey’s post hoc test, utilizing SPSS version 21.0 (SPSS Inc., Chicago, IL, USA). For all analyses, a significance threshold of *P* < 0.05 was applied.

### Reporting summary

Further information on research design is available in the Nature Research Reporting Summary linked to this article.

### Supplementary information

Supplemental Materials

Reporting Summary

## Data Availability

All data relevant to the study are included in the article or uploaded as supplementary information.
