# Supplementary material for: Polyphenol-rich diet mediates interplay between macrophage-neutrophil and gut microbiota to alleviate intestinal inflammation
Source: Cell Death Dis. 2023 Oct 9;14(10):656. doi: 10.1038/s41419-023-06190-4 (PMC10562418; doi:10.1038/s41419-023-06190-4)

**Supplementary Materials**

Table S1. Primer sequences used in RT-qPCR

| Target genes | Forward sequence (5’→3’) | Reverse sequence (5’→3’) |
| --- | --- | --- |
| **For host gene** |  |  |
| *Tnf-α* | TGGGAGTAGACAAGGTACAACCC | CATCTTCTCAAAATTCGAGTGACAA |
| *Il-1β* | CTCGCAGCAGCACATCAACAAG | GGAAGGTCCACGGGAAAGACAC |
| *Il-6* | ACCACGGCCTTCCCTACTT | CACAACTCTTTTCTCATTTCCAC |
| *Cxcl9* | GTGGTGAAATGGAAAGATCAGGGC | AAGAGAGAAATGGGTTCCCTGGAG |
| *iNos* | CAGCTGGGCTGTACAAACCTT | CATTGGAAGTGAAGCGTTTCG |
| *Il-10* | CCCTTTGCTATGGTGTCCTT | TGGTTTCTCTTCCCAAGACC |
| *Il-17a* | TCCAGAAGGCCCTCAGACTA | TGAGCTTCCCAGATCACAGA |
| *Il-22* | ATGAGTTTTTCCCTTATGGGGAC | GCTGGAAGTTGGACACCTCAA |
| *Nlrp3* | GCTAAGAAGGACCAGCCAGA | CAGCAAACCCATCCACTCTT |
| *Pkm2* | TCGCATGCAGCACCTGATT | CCTCGAATAGC TGCAAGTGGTA |
| *Hk2* | ATGCGTAATGTGGAACTGGTG | GCTGATCATCTTCTCAAACCTCTG |
| *Pdk1* | CCACTGAGGAAGA TCGACAGAC | AGAGGCGTGATATGGGCAATCC |
| *Ldha* | CGGTTCCGTTACCTGATGGG | ACCACCTGCTTGTGAACCTC |
| *Glut1* | TAGAGTGACGATCTGAGCTACGG | ACCAGTGTTATAGCCGAACYGC |
| *Occludin* | CAGCCTCGGTACAGCAGCAAT | ATAGTGGTCAGGGTCCGTCCTC |
| *Claudin-1* | AGCTGTGCATGGCCTCTTGT | CCAATGTCAATGGCAACACCCT |
| *ZO-1* | CGGAACTATGACCATCGCCTAC | CTTCGGGATGTTGTCTGGAGTC |
| *β-actin* | TGGAATCCTGTGGCATCCATGAAAC | TAAAACGCAGCTCAGTAACAGTCCG |
| *Gapdh* | AACTTTGGCATTGTGGAAGG | ACACATTGGGGGTAGGAACA |
| **For bacteria** |  |  |
| Total bacteria | GTGSTGCAYGGYYGTCGTCA | ACGTCRTCCMCNCCTTCCTC |

**Supplementary figure legends**

**Fig. S1** **Verification of depletion of the intestinal macrophage and microbiota in mice**. Mice were intraperitoneally injected of 200 μL clodronate liposomes on 2 days before 3% DSS treatment, and also on day 1 and day 4 during DSS treatment. **A** Flow cytometry of F4/80+ macrophage populations from clodronate liposomes-treated mice at day 8 after DSS challenge. Mice were intraperitoneally injected of 200 μL clodronate liposomes on 2 days before 3% DSS treatment, and also on day 1 and day 4 during DSS treatment. Mice received antibiotics in the drinking water for two weeks were orally inoculated with 0.2 mL of fecal microbial suspension from individual healthy donors once every other day for 4 weeks. **B** The concentration of fecal microbial DNA in mice after transplanted with the feces. **C** The genome copies the number of total bacteria in the feces of mice after transplanted with the feces. Data represent means ± SD of three separate experiments. Statistics was performed with unpaired two- sided Student’s *t* test or one-way ANOVA, followed by Tukey’s multiple comparison test. ***P* < 0.01.

**Fig. S2 Macrophage ablation altered the microbiota composition of DSS colitis mice.** Mice were intraperitoneally injected with 200 μL clodronate-loaded liposomes three times every two days before giving 3% DSS in drinking water. **A** LEfse of α-diversity and **B** PCoA of β-diversity as measured by Bray-Curtis dissimilarity. **C, D** Mean relative abundance at phylum level, and genus level of the gut microbiota among groups. All genera with relative abundance < 0.1% are reported together and labeled as “others”.

**Fig. S3 Phenolic acids reduce the markers of systemic and colonic inflammation. A-F** Secretion of TNF-α, IL-1β and IL-6 in serum and colon homogenates. **G** The mRNA expression of M1-characterized *Tnf-a*, *Il-1β*, *Il-6*, *Nos-2*, and *Cxcl9* in the colon from phenolic acid-administrated mice at day 8 after DSS challenge. Data represent means ± SD of three separate experiments. Statistics was performed with one-way ANOVA, followed by Tukey’s multiple comparison test. **P* < 0.05, ***P* < 0.01.

**Fig. S4** **Chlorogenic acid are capable of alleviating intestinal barrier defects in Caco2 cultured with Nlrp3-WT, but not Nlrp3-KD, macrophages.** Mice were intraperitoneally injected with 10 mg/kg CY-09 sodium, and phenolic acid was given to mice by oral gavage on day 3 of the DSS colitis model. **A** The mRNA expression of *Nlrp3* in the colon from chlorogenic acid-administrated mice after DSS challenge. **B** The mRNA level of NLRP3 in the colon homogenates of CY-09-treated mice and control littermates. **C** RT-qPCR analysis of *Nlrp3* mRNA level in NLRP3-WT and NLRP3-KD RAW264.7 macrophages. **D** Caco2 cells were treated with CGA before stimulating with LPS (1 μg/mL) for 12 h, and then co-cultured with *Nlrp3*-WT or *Nlrp3*-KD RAW264.7 macrophages, respectively. **E** TEER of Caco2 cells when co-cultured with macrophages for up to 10, 20 and 30 h. **F, G** TNF-α and IL-6 of Caco2 cells were also assayed with or without 12 h co-culture with macrophages. Data represent means ± SD of three separate experiments. Statistics was performed with unpaired two- sided Student’s *t* test or one-way ANOVA, followed by Tukey’s multiple comparison test. **P* < 0.05, ***P* < 0.01.

**Fig. S5** **Chlorogenic acid maintains mitochondrial function dependent upon PKM2.** RAW264.7 cells were transfected with control shRNA or PKM2 shRNA for 24 h, and then treated with CGA for 6 h before stimulating with LPS (1 μg/mL). **A** Effect of CGA on cell viability in PKM2-WT and PKM2-KD macrophages. **B** RT-qPCR analysis of *Pkm2* mRNA level in PKM2-WT and PKM2-KD macrophages. **C, D** Mitochondrial function was determined as mitochondrial membrane potential (ΔΨ m) and MitoSOX level. Data represent means ± SD of three separate experiments. Statistics was performed with unpaired two- sided Student’s *t* test. **P* < 0.05, ***P* < 0.01. MMP, mitochondrial membrane potential.

**Fig. S6** **Ferulic acid was able to induce less infiltration of neutrophils during colitis.** **A** Flow cytometry of CD11b+Gr-1+ neutrophil populations from ferulic acid-administrated mice at day 8 after DSS challenge. **B** Neutrophil-producing cytokines *Il-17* and *Il-22* mRNA induction in the colon. Data represent means ± SD of three separate experiments. Statistics was performed with one-way ANOVA, followed by Tukey’s multiple comparison test. **P* < 0.05, ***P* < 0.01.

**Fig. S7 Verification of depletion of the neutrophils in mice**. Mice were intraperitoneally injected of 200 μg anti-Ly6G once every three days for three times. Flow cytometry of Ly6G+ neutrophil populations from anti-Ly6G -treated mice. Data represent means ± SD of three separate experiments. Statistics was performed with unpaired two- sided Student’s *t* test. ***P* < 0.01.

**Fig. S8 Neither caffeic acid nor ellagic acid prevent colitis severity in microbiota-depleted mice.** **A** Schematic outline of the experimental design. **B, C** Colitis severity was measured as DAI, and colon length (n=8). **D, E** RT-qPCR analysis of *Tnf-α* and *Il-6* mRNA levels from CA- and EA-gavaged mice following antibiotics treatment. Data represent means ± SD of three separate experiments. Statistics was performed with Statistics was performed with unpaired two-sided Student’s *t* test. **P* < 0.05, ***P* < 0.01.

**Fig. S9 Urolithin A relieves the barrier defects in Caco2 cells induced by LPS.** Caco2 cells were treated with UroA for 6 h before stimulating with LPS (1 μg/mL). **A** TEER of Caco2 cells. **B** RT-qPCR analysis of Occludin, Claudin-4 and ZO-1 mRNA levels in LPI-challenged Caco2 cells. Data represent means ± SD of three separate experiments. Data represent means ± SD of three separate experiments. Statistics was performed with one-way ANOVA, followed by Tukey’s multiple comparison test. **P* < 0.05, ***P* < 0.01.

**Fig. S10 Urolithin A induces the reduction of ILC3s number during colitis.** Mice were intraperitoneally injected of 100 μg anti-Ly6G once every two days for twice. **A** Flow cytometry of IL22+ positive populations from anti-Il22-treated mice. **B** The percentage of RORγt+IL22+ innate lymophoid cells was detected using flow cytometry. Data represent means ± SD of three separate experiments. Statistics was performed with one-way ANOVA, followed by unpaired two-sided Student’s *t* test or Tukey’s multiple comparison test. **P* < 0.05, ***P* < 0.01.

Figure S1


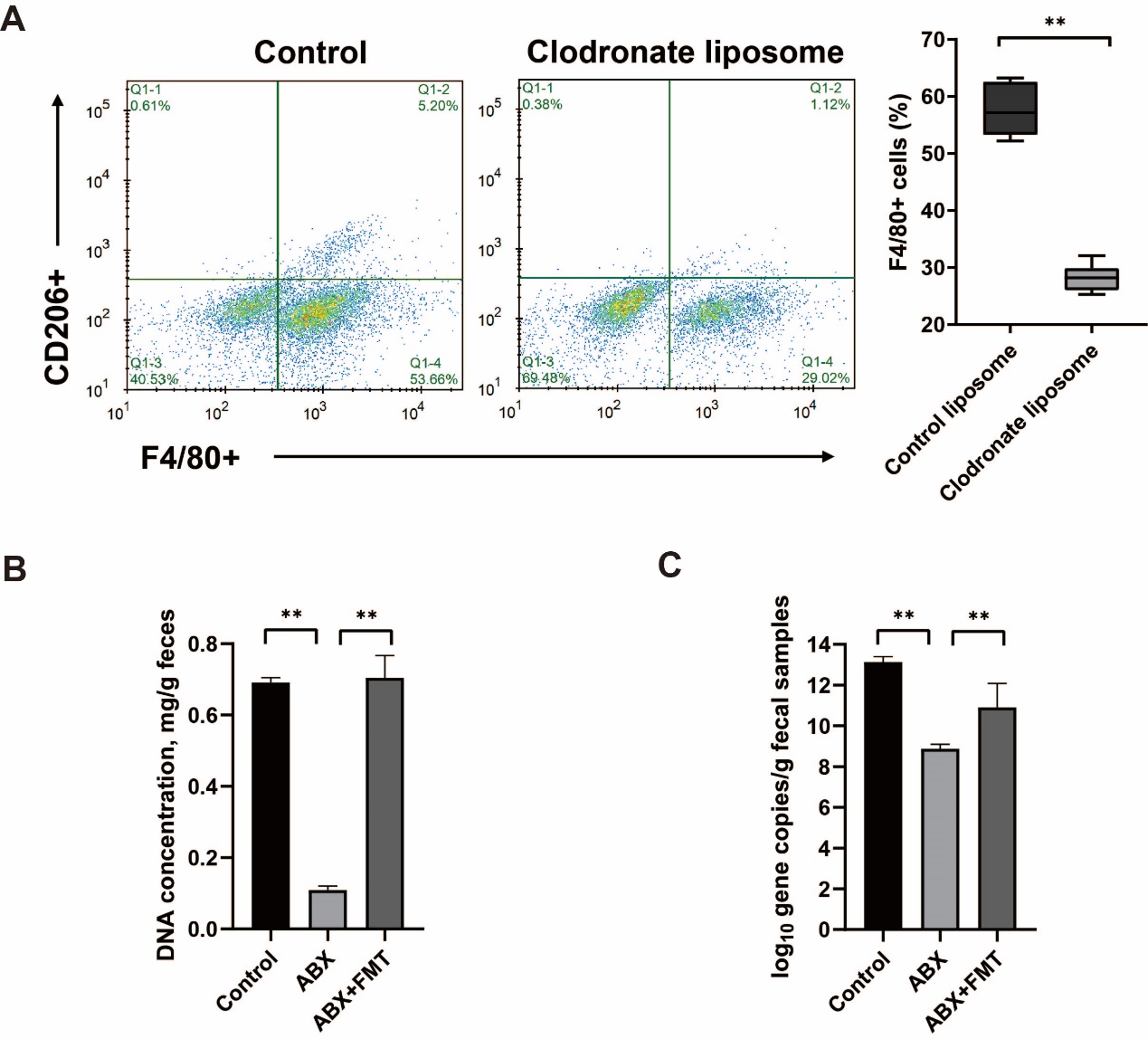


Figure S2


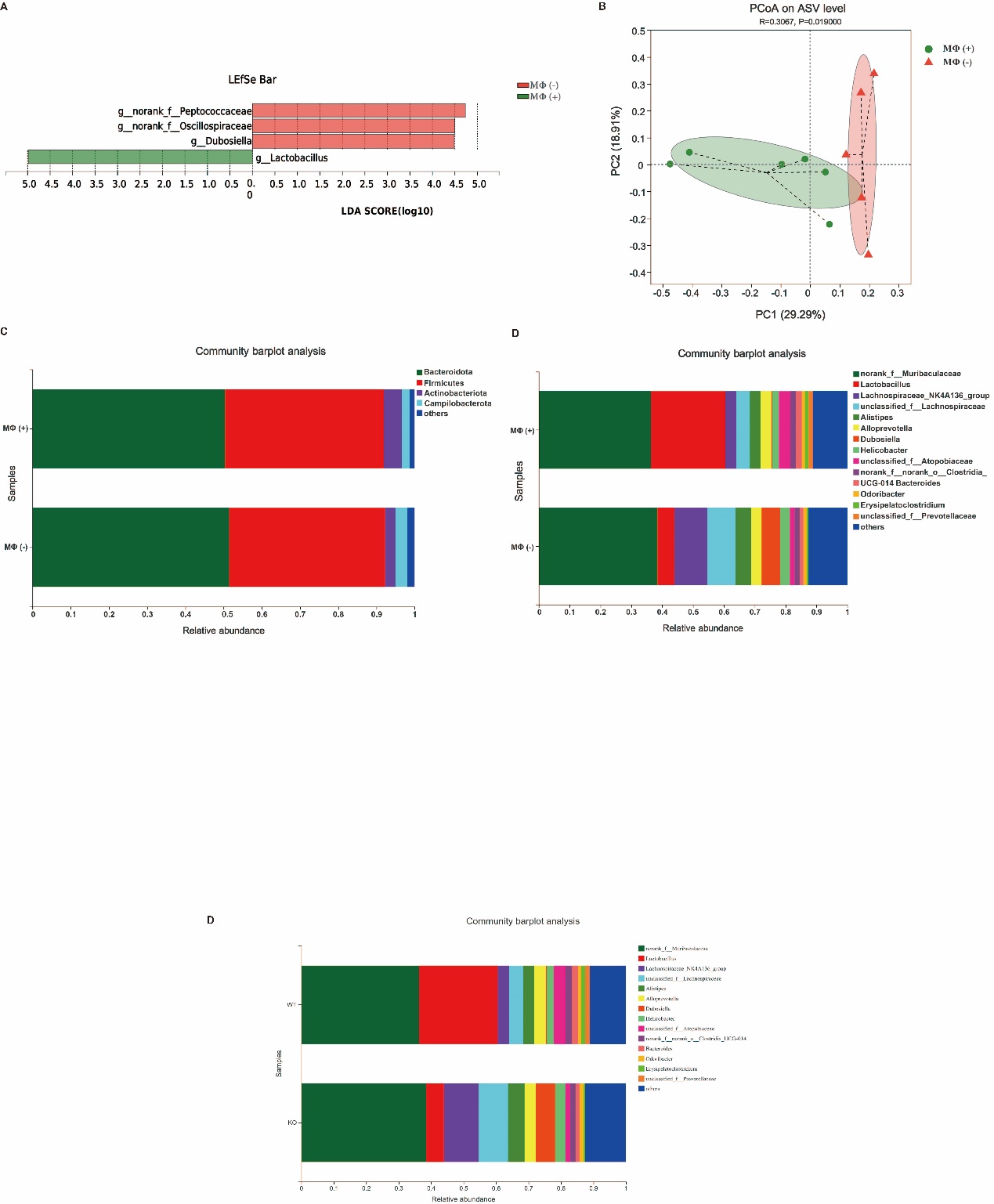


Figure S3


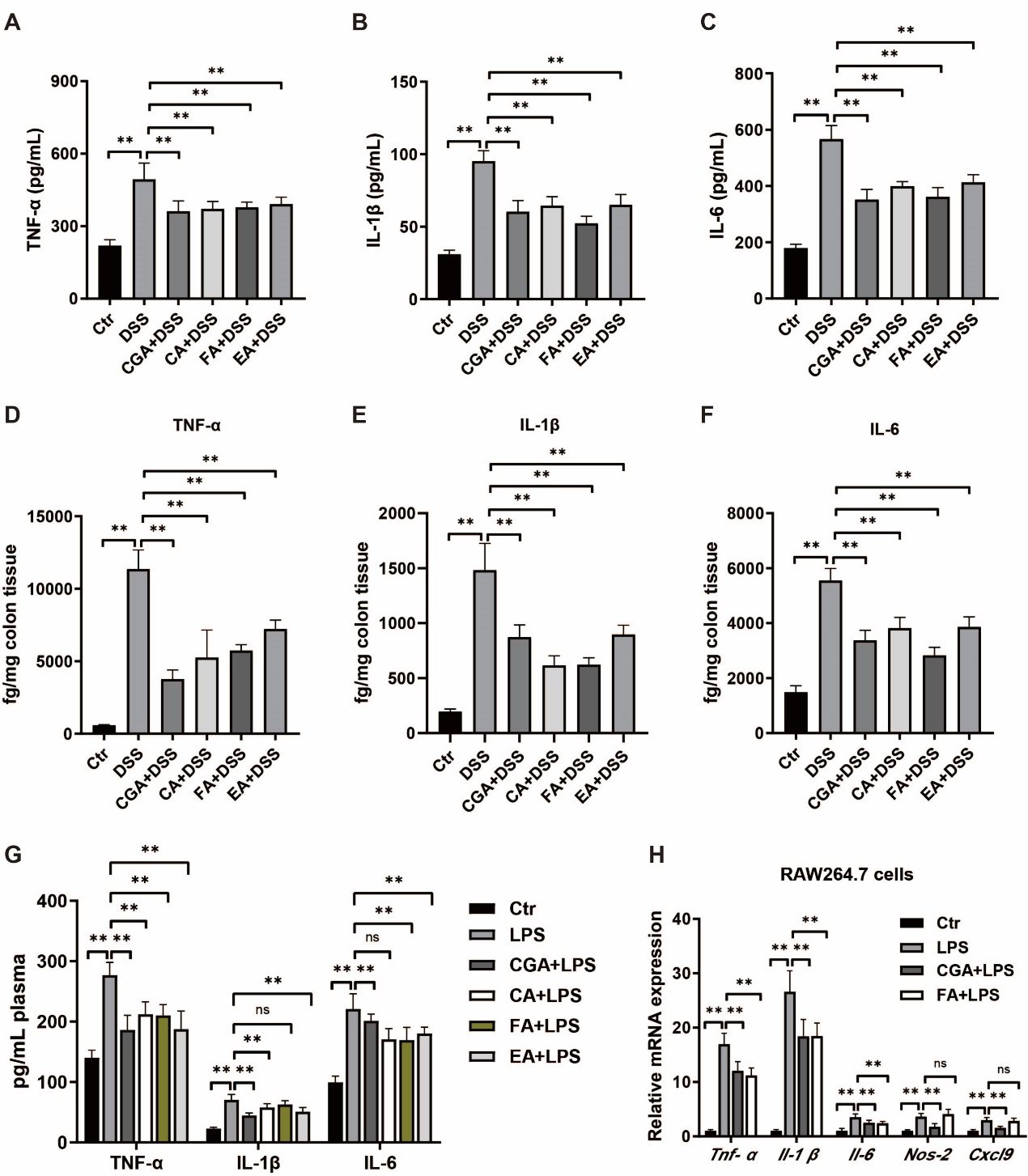


Figure S4


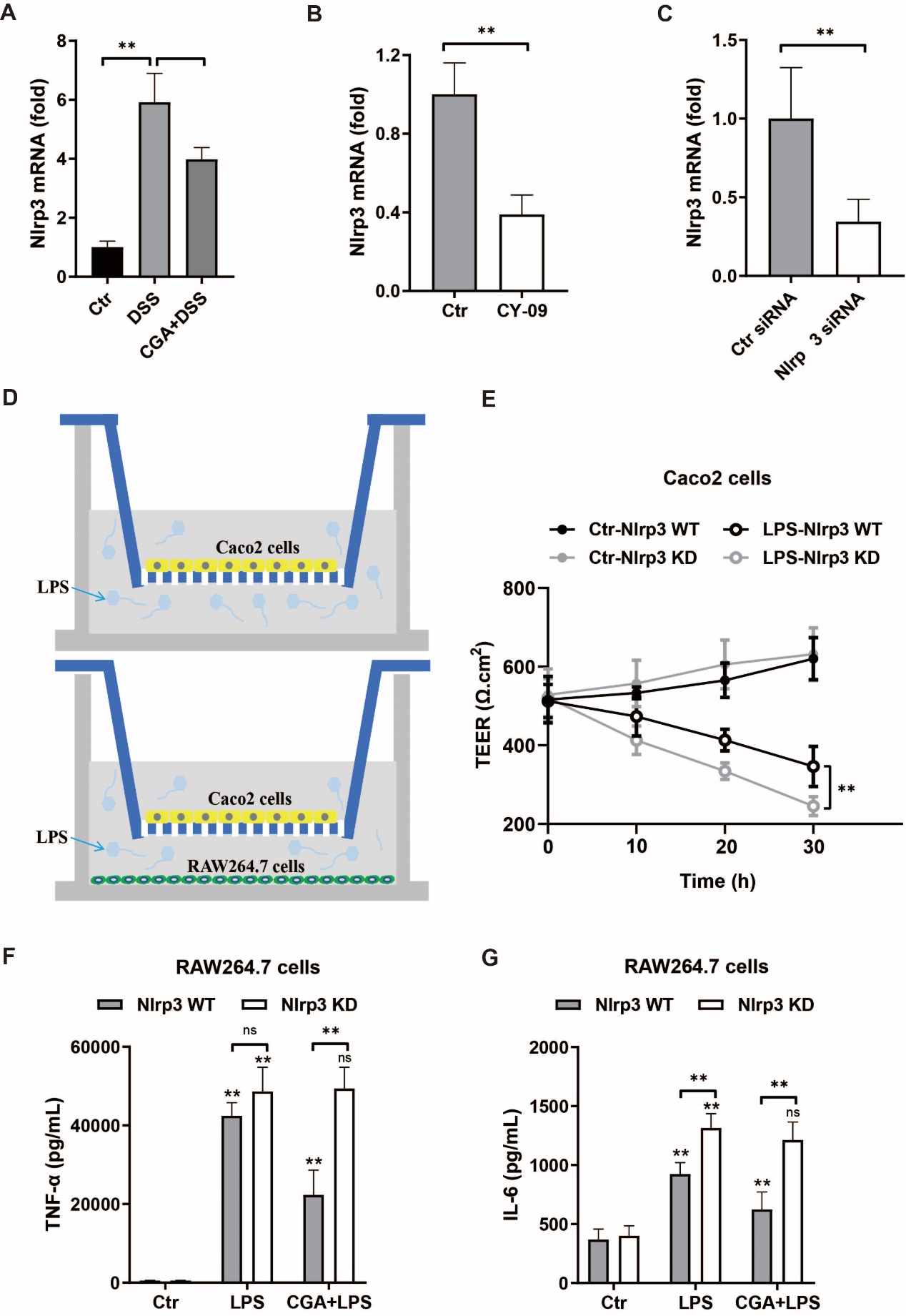


Figure S5


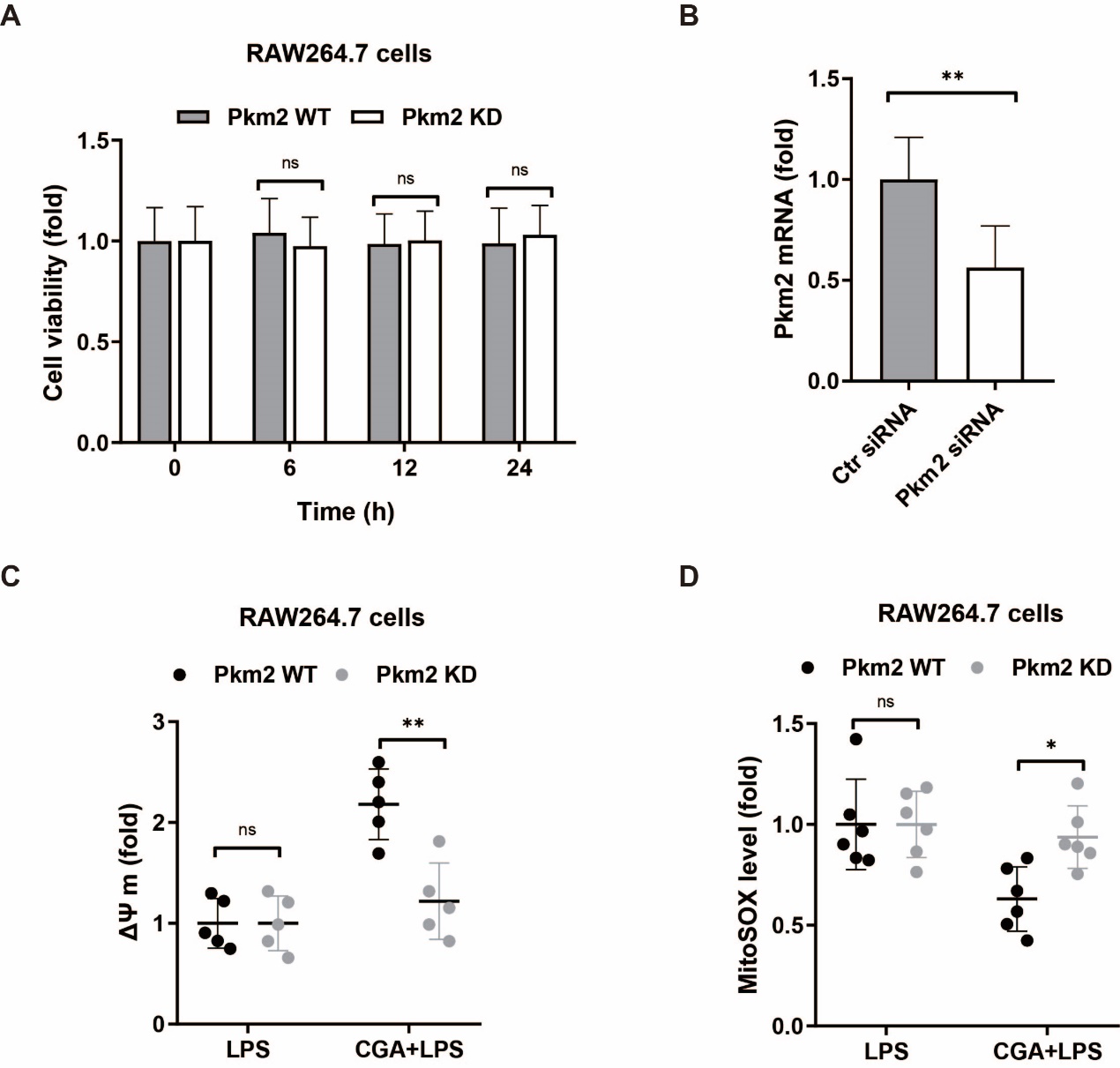


Figure S6


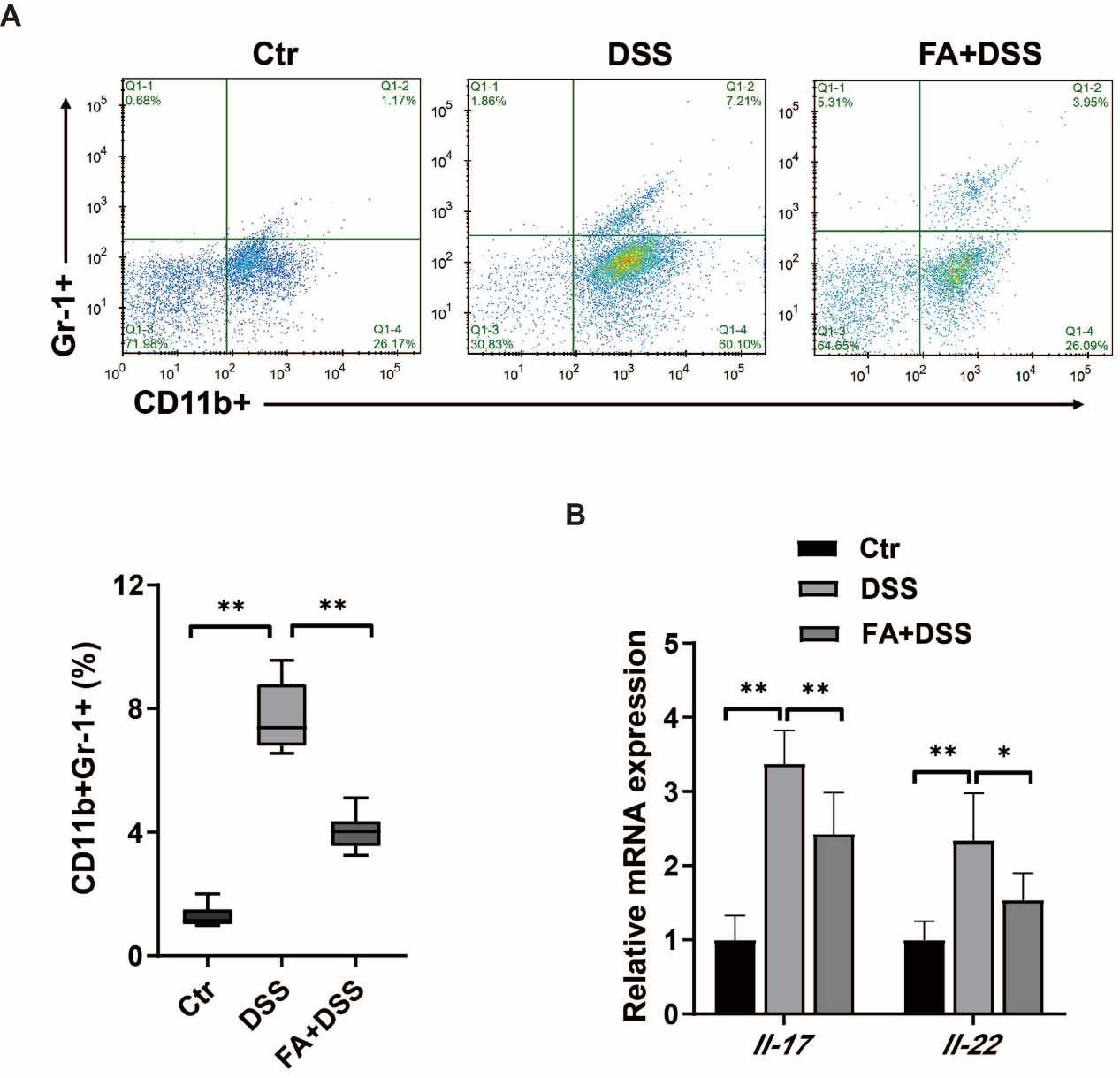


Figure S7


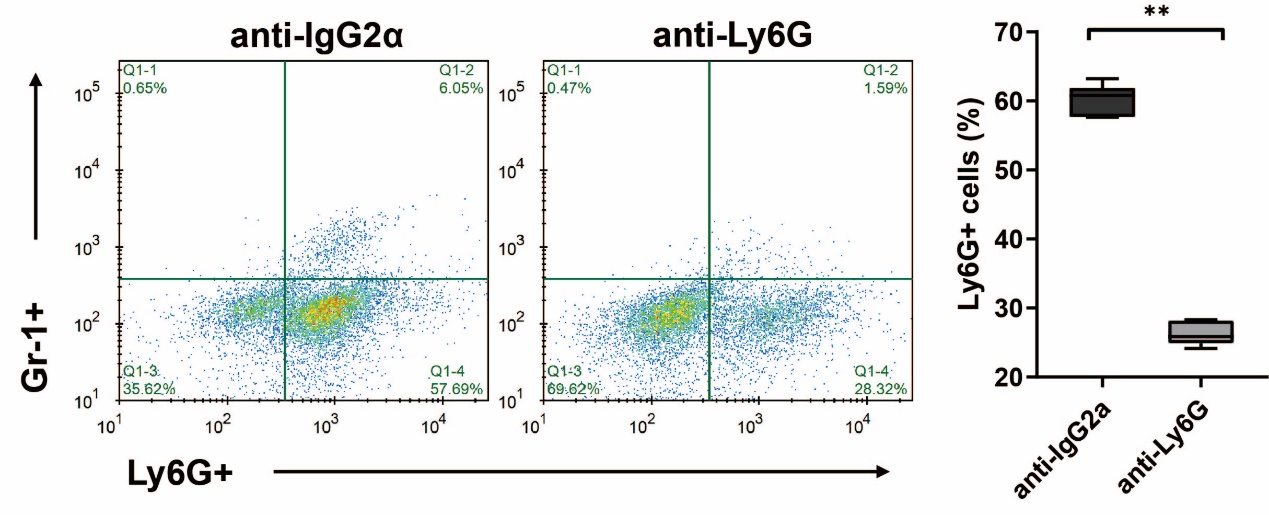


Figure S8


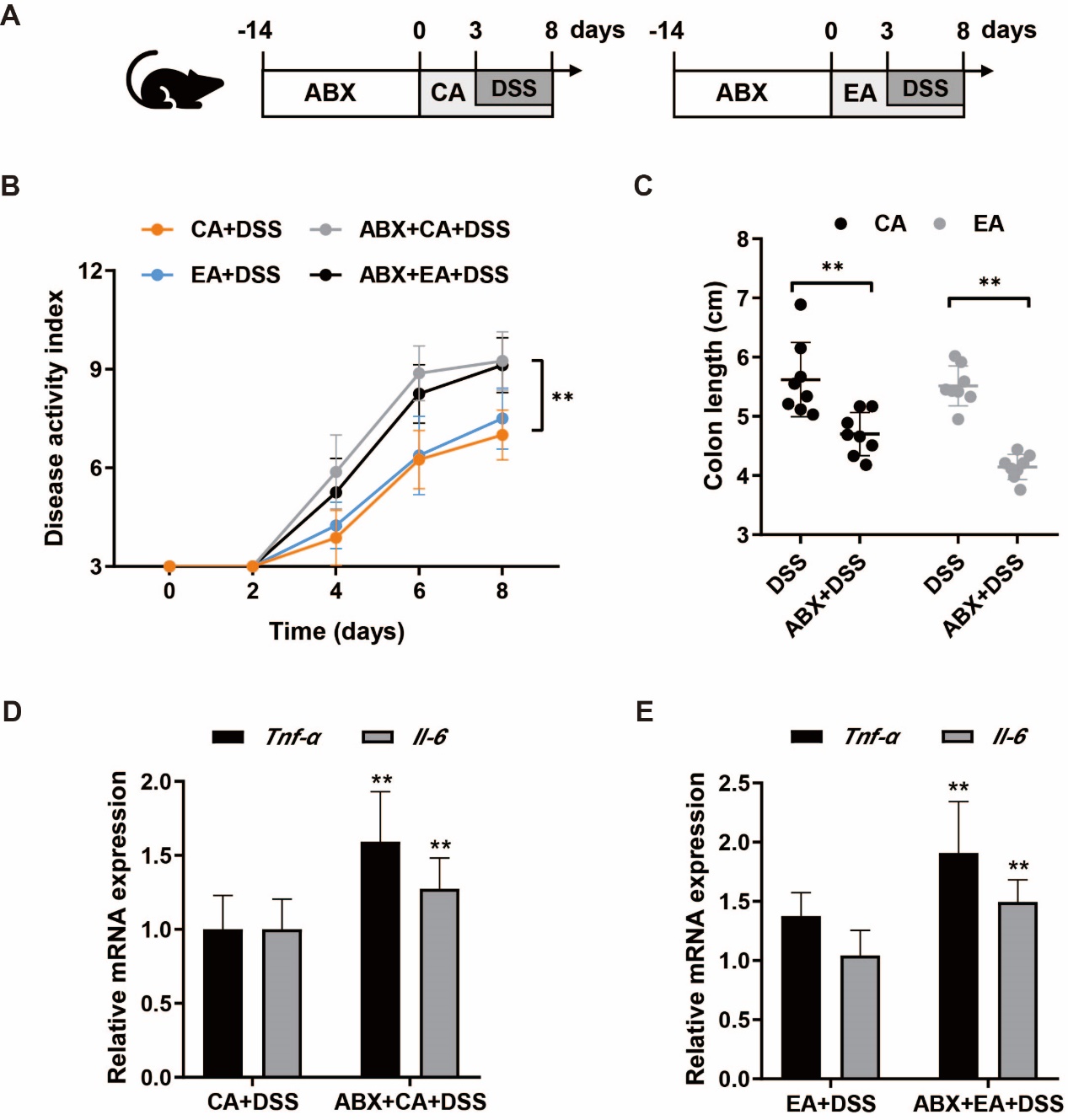


Figure S9


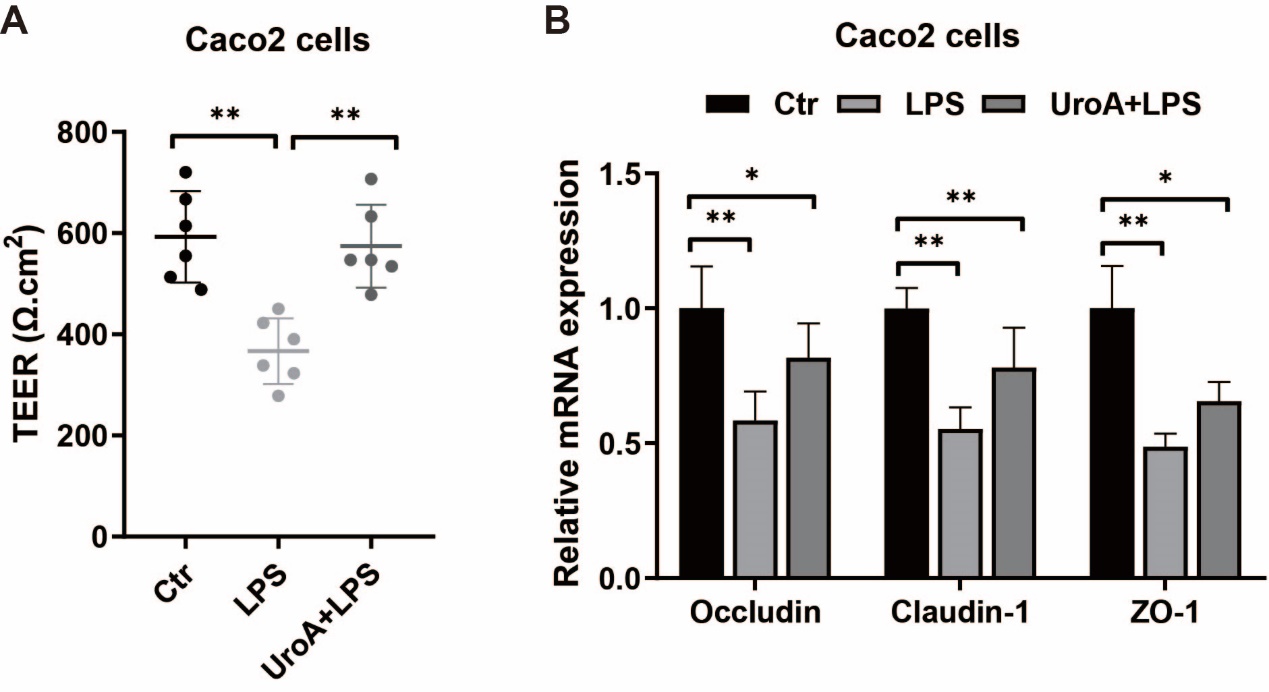


Figure S10


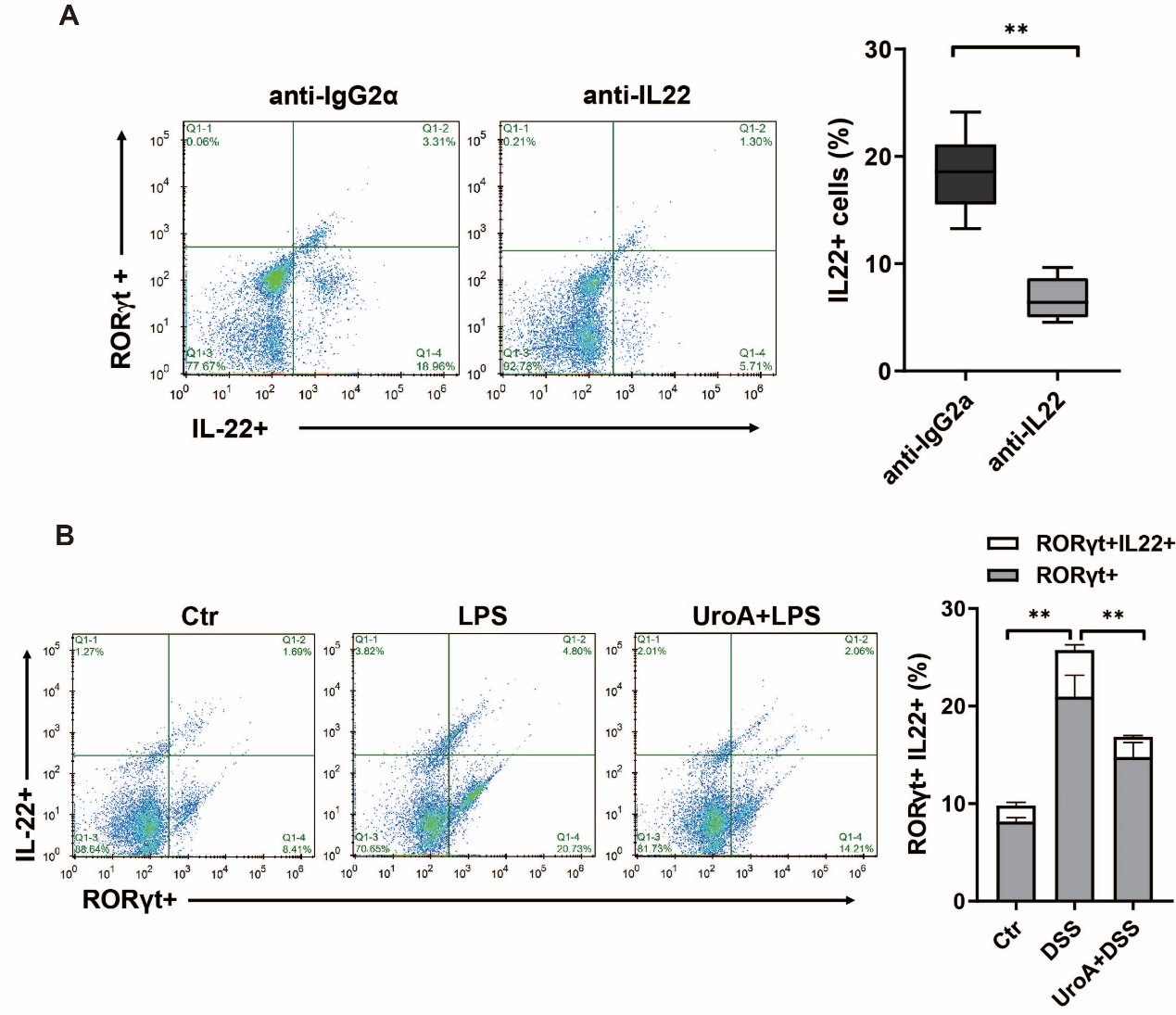

Supplement: Supplementary file 1 — Supplemental Materials [file 41419_2023_6190_MOESM1_ESM.docx]
